# Study on the Static and Dynamic Tensile Behavior of Epoxy Composites Reinforced with Nano-Alumina

**DOI:** 10.3390/polym18151861

**Published:** 2026-07-29

**Authors:** Liwei Zhang, Jinchao Qiao, Jinzhu Li

**Affiliations:** State Key Laboratory of Explosion Science and Safety Protection, Beijing Institute of Technology, Beijing 100081, China

**Keywords:** nano-Al_2_O_3_/epoxy composites, quasi-static tensile behavior, dynamic tensile behavior, strain rate sensitivity, particle agglomeration, fracture toughness, interfacial adhesion

## Abstract

Epoxy resins suffer from inherent brittleness, limiting their reliability in impact-resistant structures. This study addresses the dispersion–performance trade-off in nano-alumina (Al_2_O_3_)/epoxy composites by fabricating specimens with 0–15 wt% filler loadings using an optimized ultrasonic-mechanical dispersion strategy. Quasi-static and dynamic tensile behaviors (600–1600 s^−1^) were evaluated using a universal tester and a split Hopkinson tensile bar (SHTB) system equipped with high-sensitivity semiconductor strain gauges. Results identify a critical agglomeration threshold at 3 wt%. The 1 wt% composite exhibited optimal quasi-static strength (44.76 ± 0.25 MPa), a 4.0% improvement over the neat epoxy (43.04 ± 0.33 MPa). While all composites showed positive strain-rate sensitivity, nano-Al_2_O_3_ incorporation generally reduced dynamic strength, except for the 5 wt% composite at intermediate rates. Notably, the 15 wt% composite recovered to 78.49 ± 0.48 MPa at 1600 s^−1^ due to high-rate energy dissipation mechanisms. Microstructural analysis revealed a transition from brittle cleavage to a hybrid fracture mode dominated by microvoids and localized plastic tearing. This work quantitatively defines the optimal loading window for nano-Al_2_O_3_/epoxy composites in protective engineering.

## 1. Introduction

Epoxy resin (EP) is widely recognized as a high-performance thermosetting polymer matrix [1,2], extensively utilized in aerospace [3,4,5,6], automotive manufacturing [7,8,9,10,11], electronic packaging [12,13,14,15,16,17,18], and civil engineering [19,20,21,22,23,24,25] due to its exceptional adhesive properties [26,27,28,29,30], superior chemical stability [31], low curing shrinkage [32], and excellent processability [33]. In practical service environments, epoxy-based structural components are frequently subjected to complex mechanical loads. Particularly under extreme conditions such as impact or explosion, materials exhibit significantly different mechanical responses compared to quasi-static conditions, often demonstrating a higher propensity for brittle failure [34]. Consequently, a comprehensive understanding of the static and dynamic mechanical behaviors of epoxy resins and their composites is crucial for advanced structural design and safety assessment.

To address the inherent brittleness and insufficient impact resistance of pure epoxy resin, the incorporation of nanofillers to fabricate high-performance nanocomposites has emerged as a prominent research focus in recent years. Among various reinforcing phases, nano-alumina (Al_2_O_3_) is regarded as an ideal candidate for enhancing polymer matrices owing to its high elastic modulus, high strength, excellent thermal resistance, chemical stability, and relatively moderate cost [35,36,37]. However, the strong tendency of nanoparticles to agglomerate poses significant challenges to achieving uniform dispersion within the epoxy matrix, and the interfacial interactions between the fillers and the matrix play a decisive role in the final macroscopic mechanical performance, particularly under dynamic loading.

Compared with other candidate reinforcing fillers, nano-Al_2_O_3_ offers a distinctive combination of properties that make it particularly attractive for epoxy-matrix composites. Metallic fillers such as Al and Cu, as well as carbon-based fillers such as carbon nanotubes and graphene, can substantially improve thermal conductivity, yet their high or non-negligible electrical conductivity undermines dielectric performance and renders them unsuitable for electrically insulating systems [38]. In contrast, α-Al_2_O_3_ combines a relatively high intrinsic thermal conductivity (≈ 30 W/(m·K)), a wide band gap (4.3 eV) and a high volume resistivity on the order of 10^14^ Ω·cm, thereby simultaneously satisfying the thermal-management and electrical-insulation requirements of electronic packaging and protective engineering [39]. Among ceramic alternatives, silica (SiO_2_) is inexpensive but its thermal conductivity is only 1–1.5 W/(m·K); boron nitride and aluminum nitride possess higher intrinsic thermal conductivities, yet their significantly higher cost and poor dispersibility in polymer matrices limit their practical deployment [39]. Furthermore, appropriately dispersed nano-Al_2_O_3_ enhances the fracture toughness of epoxy resins through crack pinning, crack deflection, particle pull-out and localized plastic yielding of the matrix [40,41], while silane surface functionalization further improves particle–matrix interfacial adhesion and suppresses agglomeration, thereby enabling more effective load transfer [42]. These combined attributes—balanced thermal/electrical properties, competitive cost, toughening capability and interfacial compatibility—distinguish nano-Al_2_O_3_ from alternative reinforcing fillers and motivate its selection in the present study.

Tensile loading of polymeric materials involves a sequential evolution from elastic to plastic deformation, the mechanistic understanding of which is essential for interpreting mechanical test data. In the elastic regime, the polymer network stores recoverable strain energy through bond stretching and conformational rearrangements of molecular chains, with stress increasing nearly linearly with strain [43]. As the applied stress exceeds the yield point, the material enters a plastic deformation stage governed by molecular-level mechanisms such as chain uncoiling, segmental motion, shear transformation zones, and the nucleation and propagation of microvoids and crazes [44]. For crosslinked thermosetting polymers such as epoxy resin, recent multiscale constitutive studies have demonstrated that the elastic–plastic response is strongly coupled with strain rate, temperature, and crosslinking density, producing characteristic yielding and post-yield softening under both quasi-static and dynamic conditions [45]. The competition between these deformation modes dictates whether the material fails in a ductile or brittle manner and is strongly modulated by loading rate and temperature [44,45].

The distinction between quasi-static and dynamic tensile behavior is therefore central to the engineering application of epoxy-based composites. Under quasi-static loading (typically 10^−4^–10^−2^ s^−1^), epoxy resins exhibit pronounced viscoelastic–plastic responses, with measurable yielding and necking prior to fracture [46]. Under high-rate loading (10^2^–10^3^ s^−1^), the available time for stress relaxation and molecular rearrangement is drastically reduced, which generally suppresses plastic dissipation and promotes brittle failure, while simultaneously elevating strength and modulus—a phenomenon known as positive strain-rate sensitivity [47]. Systematic experimental studies have confirmed that both the tensile strength and elastic modulus of epoxy resins increase monotonically with strain rate over wide ranges; for example, Kumar et al. [46] reported strain-rate-dependent stiffening and strengthening of a bisphenol-A epoxy from 10^−4^ s^−1^ up to ~2800 s^−1^, while Liu et al. [47] observed analogous trends in TDE86 epoxy under quasi-static and dynamic tensile loading. Capturing this rate-dependent transition is indispensable for predicting material failure under realistic impact scenarios.

Although extensive studies have been conducted on the mechanical properties of epoxy composites reinforced with carbon nanotubes [48,49,50], graphene [51,52,53], silica nanoparticles [54,55,56], halloysite nanotubes (HNT) [57], or carboxyl-terminated butadiene-acrylonitrile (CTBN) rubber [58], research specifically focusing on the tensile behavior of nano-Al_2_O_3_ reinforced epoxy composites under a wide range of strain rates remains insufficient. Existing literature indicates that while nano-Al_2_O_3_ can effectively improve the stiffness and toughness of polymers such as polyether sulfone and phenolic resins through mechanisms like crack deflection and plastic deformation [59,60,61], its reinforcing efficiency in epoxy matrices under high-strain-rate tension is not yet fully understood. Furthermore, most current dynamic mechanical investigations are limited to compression tests using the Split Hopkinson Pressure Bar (SHPB), while studies on dynamic tensile properties—which are critical for predicting material failure in actual stress states—are relatively scarce. The lack of systematic experimental data regarding the effects of filler content gradients and dispersion quality on the damage evolution and energy absorption mechanisms under dynamic tension hinders the quantitative design and optimization of such materials for anti-impact applications.

Among the fillers previously studied in epoxy systems, carbon nanotubes and graphene typically require only very low loadings to produce substantial quasi-static tensile improvements, but their high or non-negligible electrical conductivity restricts them to electrically conducting applications, and their high-aspect-ratio or two-dimensional geometries make homogeneous dispersion progressively more difficult as loading increases. Silica nanoparticles offer a low-cost route to toughening, yet their intrinsically low thermal conductivity limits their suitability where thermal management is required. In contrast, the nano-Al_2_O_3_/epoxy composites investigated here combine electrical insulation, relatively high thermal conductivity, and moderate cost, while the present work—unlike most prior studies that focus on compression—provides systematic dynamic tensile data across a 600–1600 s^−1^ strain-rate range, thereby clarifying both the reinforcement potential and the agglomeration-induced limitations of this filler in tension-dominated impact scenarios.

To bridge this gap, this study aims to systematically investigate the static and dynamic tensile properties of nano-Al_2_O_3_-reinforced epoxy resin composites. An optimized direct dispersion method was employed to prepare composite specimens with six different nano-Al_2_O_3_ mass fractions (0 wt%, 1 wt%, 3 wt%, 5 wt%, 10 wt%, and 15 wt%). To accommodate the varying dispersion difficulties across these content gradients, differentiated processing parameters were implemented during the preparation process. Quasi-static tensile tests were performed using a universal testing machine, while high-strain-rate tensile tests were conducted using Split Hopkinson tensile bars (SHTB). The primary objective of this work is to reveal the regulatory effect of nano-Al_2_O_3_ content on the tensile strength, fracture toughness, and energy absorption capacity of the composites under both quasi-static and dynamic loading conditions. Additionally, scanning electron microscopy (SEM) was utilized to characterize the microscopic failure modes and interfacial interactions. By elucidating the micro-mechanisms of reinforcement and toughening—such as nanoparticle debonding, crack bridging, and matrix shear banding—this study seeks to provide theoretical and experimental foundations for the engineering application of nano-Al_2_O_3_/epoxy composites in impact-resistant protection structures.

## 2. Materials and Methods

### 2.1. Materials and Specimen Preparation

In this study, 3M DP460 two-part toughened epoxy structural adhesive was selected as the matrix material. Component A (resin) appears as a white paste, while Component B (hardener) is an amber liquid. The two components were mixed at a volume ratio of 2:1 and cured to a beige solid. The operational time of the adhesive is approximately 60 min at 22 °C, reaching handling strength within 4 h and achieving initial cure after 24 h. The reinforcing filler used was nano-Al_2_O_3_ powder, which presents as a white powder with a particle size of ≤ 500 nm and a high purity of 99.9%, featuring a uniform particle size distribution and a clean surface free of impurities. To investigate the effect of nano-Al_2_O_3_ content on the mechanical properties of the epoxy composites, six mass fractions were designed: 0 wt%, 1 wt%, 3 wt%, 5 wt%, 10 wt%, and 15 wt%.

An optimized direct dispersion method was adopted to prepare the nano-Al_2_O_3_/epoxy composites, implementing differentiated process controls tailored to the specific content gradients. Raw materials were accurately weighed using a high-precision electronic balance according to the epoxy resin-to-hardener mass ratio of 2:1 and the predetermined filler content. To address the issue of nanoparticle agglomeration, a “stepwise” ultrasonic-mechanical synergistic dispersion strategy was employed. For low-content groups (1–3 wt%), ultrasonication was performed at 250 W for 10 min; for medium-to-high-content groups (5–15 wt%), the power was increased to 300 W and the duration extended to 15 min, with manual stirring supplemented during the process to ensure uniform dispersion. After dispersion, the mixture was mechanically stirred at 500 r/min for 5 min using an electric stirrer to guarantee homogeneity, followed by defoaming in a vacuum chamber at −0.08 MPa for 5 min to eliminate internal defects.

The degassed slurry was poured into molds and cured at a constant temperature of 25 °C. Gradient curing durations were applied based on the filler content: 24 h for low-content samples, 36 h for medium-content samples, and 48 h for high-content samples, ensuring complete cross-linking. After curing, the specimens were gently demolded, and defective samples exhibiting bubbles, cracks, or other flaws were discarded. The cured composite blanks were machined to standard dimensions using lathes and milling machines. The surfaces and edges were polished, and precise measurements were taken to control dimensional tolerances within ±0.05 mm, yielding test specimens with smooth surfaces and qualified geometries.

**Figure 1:**
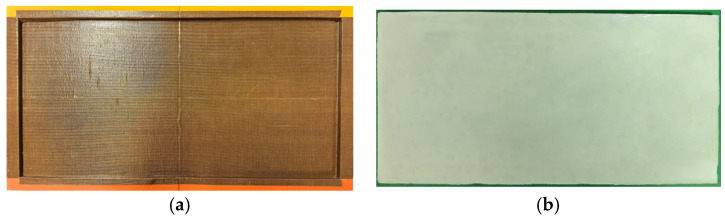
Fabrication of quasi-static tensile specimens: (**a**) 3D-printed mold; (**b**) Cured and demolded specimen blank.

Polyethylene terephthalate glycol-modified (PETG) was selected as the mold material because it is a fused deposition modeling (FDM)-compatible thermoplastic that permits the fabrication of molds with complex geometries and controlled dimensional accuracy [62]. The specimen molds were fabricated integrally using FDM 3D printing technology, as shown in Figure 1a. The well-mixed nano-Al_2_O_3_/epoxy slurry was cast into the mold cavities. After curing for 48 h, rectangular prism-shaped composite specimens were obtained, as illustrated in Figure 1b.

**Figure 2:**
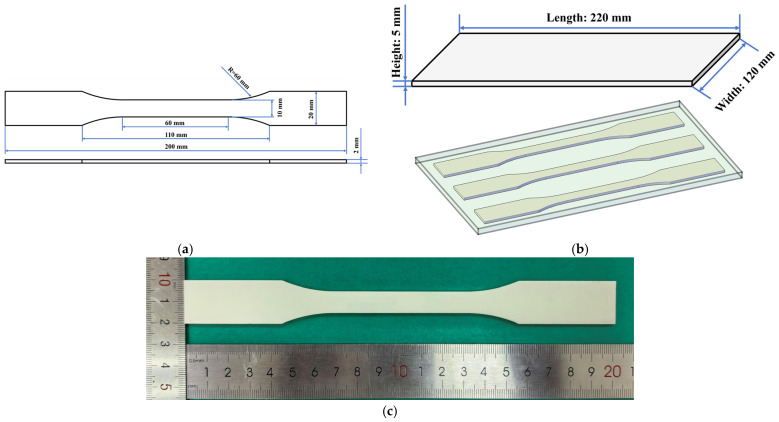
Schematic diagrams and photograph of the quasi-static tensile specimen: (**a**) Dimensional drawing; (**b**) Schematic diagram of the machining process; (**c**) Photograph of the fabricated specimen.

To prevent premature failure caused by stress concentration at the gripping ends, the quasi-static tensile specimens were designed with a dumbbell-shaped geometry featuring wider ends and a narrower gauge section. The design dimensions of the specimens used for quasi-static tensile testing are illustrated in Figure 2a. According to these specifications, the initial rectangular billets (220 mm × 120 mm × 5 mm) were machined into dumbbell-shaped specimens measuring 200 mm in total length, with a gauge section width of 10 mm and a thickness of 2 mm, as detailed in Figure 2b. The final fabricated specimens are shown in Figure 2c.

**Figure 3:**
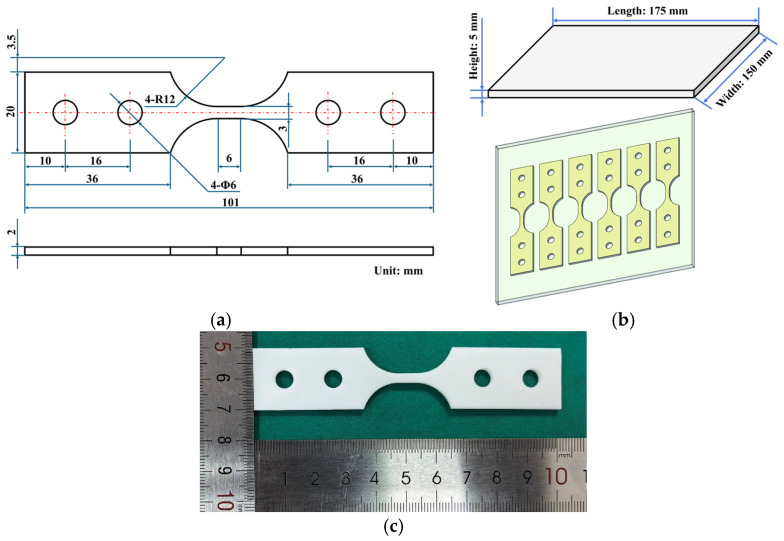
Schematic diagrams and photograph of the dynamic tensile specimen: (**a**) Dimensional drawing; (**b**) Schematic diagram of the machining process; (**c**) Photograph of the fabricated specimen.

Dynamic tensile specimens with a thickness of 2 mm, a total length of 101 mm, and a gauge section length of 6 mm were designed and fabricated. The detailed dimensions of the specimen are illustrated in Figure 3a. Based on these specifications, the initial plate blank with dimensions of 175 mm × 150 mm × 5 mm was machined to obtain the final specimens, as shown in Figure 3b. The photograph of the fabricated specimen is presented in Figure 3c.

**Figure 4:**
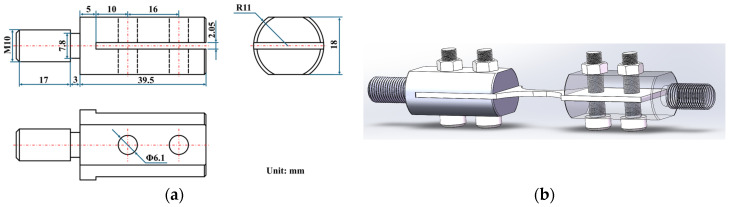
Dedicated fixtures for dynamic tensile experiments: (**a**) Dimensional drawing of the fixture; (**b**) Schematic diagram of the fixture assembled with the specimen.

In the dynamic tensile experiments, the specimens were clamped using dedicated fixtures. The structural dimensions of the fixture are illustrated in Figure 4a, which achieves reliable connection and fixation with the SHTB via threaded interfaces. The overall assembly status after mounting the specimen into the fixture is shown in Figure 4b. This fixture was specifically designed to ensure alignment accuracy and avoid eccentric loading, while providing stable clamping force through the threaded connection to minimize slippage or stress concentration. The design guarantees the centering and connection stability of the specimen during the dynamic loading process.

### 2.2. Quasi-Static Tensile Test

The quasi-static tensile tests of the nano-Al_2_O_3_ reinforced epoxy resin composites were conducted using a universal testing machine. The equipment is hydraulically driven and applies tensile loading at different strain rates through the crosshead. During the experiments, data including loading time, crosshead displacement, and applied load were automatically recorded by the data acquisition system integrated with the SAAS universal testing machine.

**Figure 5:**
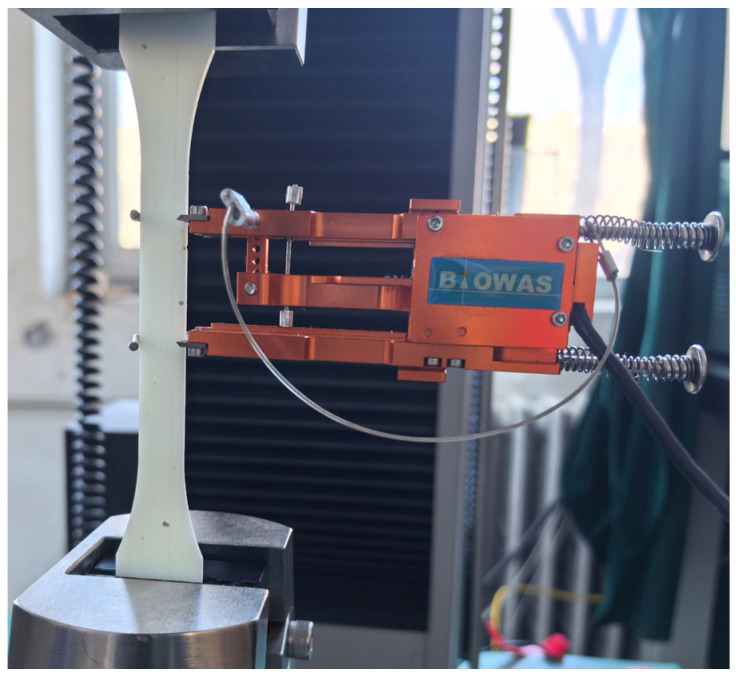
Installation of the specimen on the universal testing machine.

To investigate the effect of nano-Al_2_O_3_ content on the quasi-static tensile properties of the epoxy composites, tensile tests were performed on specimens with six different filler contents ranging from 0 wt% to 15 wt%. The experiments were carried out at two strain rates of 0.001 s^−1^ and 0.01 s^−1^, corresponding to crosshead speeds of 5 mm/min and 50 mm/min, respectively. All tests were repeated three times to ensure the reliability and repeatability of the data. Figure 5 shows the assembly status of the tensile specimen mounted on the universal testing machine.

### 2.3. Dynamic Tensile Test

**Figure 6:**
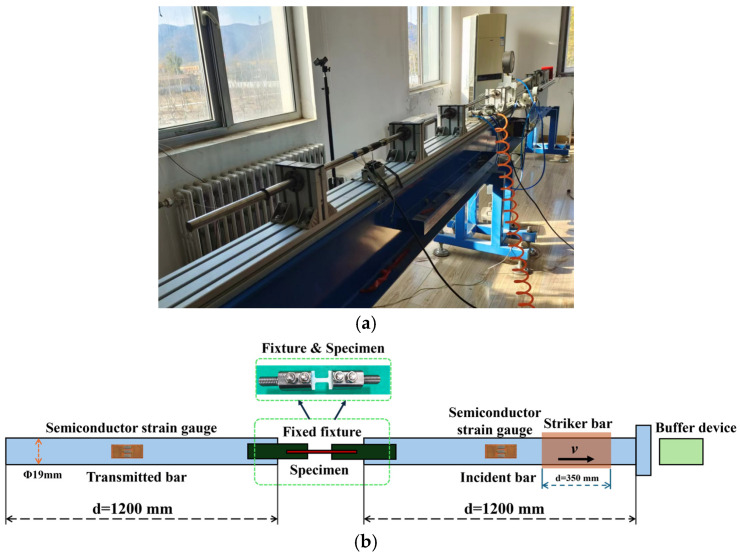
SHTB testing system: (**a**) Photograph of the experimental setup; (**b**) Schematic diagram of the working principle.

The dynamic tensile tests were performed on an SHTB system, depicted in Figure 6a. As shown in Figure 6b, upon impact of a sleeve-shaped striker on the flange at the incident bar tip, a tensile stress wave is initiated in the incident bar, thereby dynamically loading the specimen in tension.

The SHTB experiments operate on the same fundamental principles as SHPB tests, both requiring adherence to the assumptions of one-dimensional stress wave propagation and stress uniformity within the specimen. Based on the incident strain $\varepsilon_{I}\left(t\right)$, reflected strain $\varepsilon_{R}\left(t\right)$, and transmitted strain $\varepsilon_{T}\left(t\right)$ recorded by the data acquisition system, the dynamic tensile stress $\sigma \left(t\right)$, strain rate $\dot{\varepsilon }\left(t\right)$, and strain $\varepsilon \left(t\right)$ of the epoxy resin material can be calculated as follows [63,64,65]:

(1) $$\sigma \left(t\right)=\frac{E_{B}A_{B}\varepsilon_{T}\left(t\right)}{A_{S}}$$

(2) $$\dot{\varepsilon }\left(t\right)=\frac{2c_{B}\varepsilon_{R}\left(T\right)}{L}$$

(3) $$\varepsilon \left(t\right)=\frac{2C_{B}}{L}\int_{0}^{t}\varepsilon_{R}\left(t\right)dt$$

In these equations, $E_{B}$ denotes the elastic modulus of the bars, $A_{B}$ represents the cross-sectional area, $C_{B}$ is the longitudinal wave speed within the bars, L is the initial length of the specimen gauge section, and $A_{S}$ is the cross-sectional area of the specimen.

Due to the inherently low wave impedance of polymeric materials, conventional SHTB systems often yield weak transmitted signals, leading to potential measurement errors. To enhance signal quality and testing accuracy, high-sensitivity semiconductor strain gauges (Type TP3.8120, Kechuang Sensor Co., Bengbu, China) were employed to replace standard resistive strain gauges. These semiconductor gauges feature a maximum gauge factor of 110 and a measurable strain range of ±6000 µε. They effectively amplify the faint strain signals in the transmission bar, thereby significantly improving the signal-to-noise ratio and reducing measurement uncertainty. The key material parameters for the dynamic loading experiments are summarized in Table 1.

To ensure the validity of data reduction based on the one-dimensional stress wave theory (Equations (1)–(3)), it is imperative to verify that the specimen achieves a state of stress equilibrium during dynamic tensile loading, i.e., the forces at both ends of the specimen are equal. In this study, stress equilibrium was verified by comparing the loads at the two specimen–bar interfaces, derived from the incident ($\varepsilon_{I}$), reflected ($\varepsilon_{R}$), and transmitted ($\varepsilon_{T}$) strain signals. Specifically, the condition $\varepsilon_{I}+\varepsilon_{R}\approx \varepsilon_{T}$ was considered indicative of stress equilibrium and homogeneous deformation within the specimen. During data processing, only valid experimental records satisfying this equilibrium criterion throughout the primary loading phase were retained for analysis; data exhibiting severe signal oscillations or significant deviations from equilibrium were excluded.

Dynamic tensile tests were conducted on the epoxy composites with four representative nano-Al_2_O_3_ contents (0 wt%, 5 wt%, 10 wt%, and 15 wt%) using the SHTB system. Loading at different strain rates was achieved by varying the gas pressure driving the projectile. Three pressure levels—0.095 MPa, 0.12 MPa, and 0.15 MPa—were employed, and the corresponding testing conditions are summarized in Table 2.

## 3. Results and Discussion

### 3.1. Analysis of Quasi-Static Tensile Test Results

**Figure 7:**
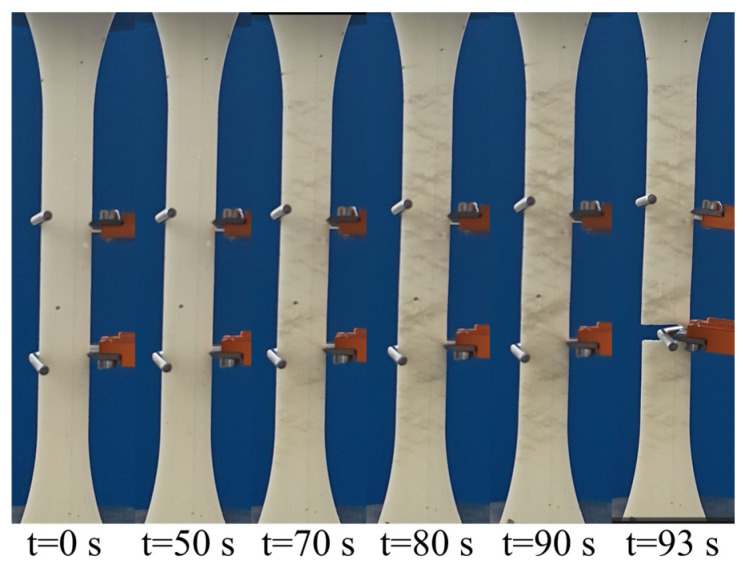
Tensile-to-fracture process of the specimen at a strain rate of 0.001 s^−1^.

Figure 7 illustrates the fracture process of the nano-Al_2_O_3_/epoxy composites under quasi-static tensile loading, as captured by a camera. From *t* = 0 to 50 s, the specimen undergoes an elastic deformation stage, characterized by uniform elastic elongation without visible damage. Between *t* = 50 and 70 s, the material enters a plastic deformation stage, during which uniformly distributed white moiré-like damage bands initiate and propagate across the specimen surface, accompanied by significant plastic elongation in the gauge section. From *t* = 70 to 90 s, the process transitions to the necking stage, where damage localizes, leading to pronounced necking and the initiation of the main crack within the necked region. Finally, between *t* = 90 and 93 s, the specimen experiences rapid fracture; the main crack propagates unstably, culminating in complete separation within 3 s.

**Figure 8:**
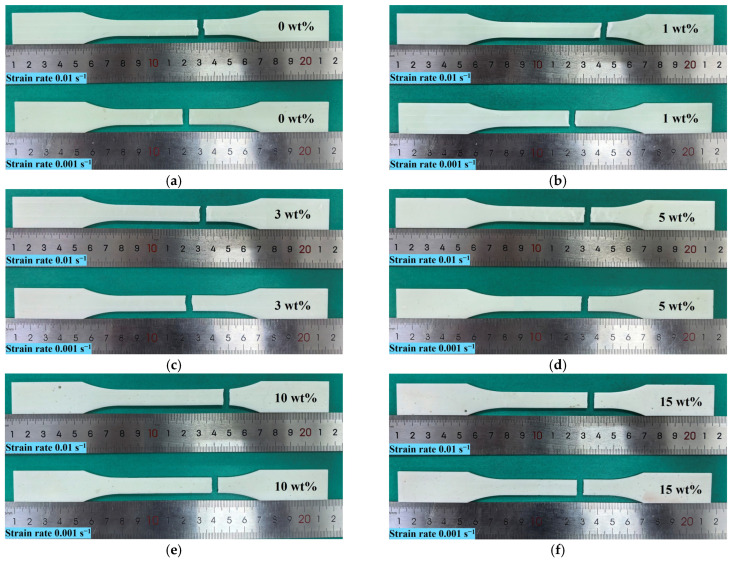
Macroscopic morphologies of tensile damage in nano-Al_2_O_3_/epoxy composites under two strain rates: (**a**) Epoxy composite with 0 wt% nano-Al_2_O_3_ (Neat epoxy resin); (**b**) Epoxy composite with 1 wt% nano-Al_2_O_3_; (**c**) Epoxy composite with 3 wt% nano-Al_2_O_3_; (**d**) Epoxy composite with 5 wt% nano-Al_2_O_3_; (**e**) Epoxy composite with 10 wt% nano-Al_2_O_3_; (**f**) Epoxy composite with 15 wt% nano-Al_2_O_3_.

Figure 8 presents the macroscopic post-fracture morphologies of nano-Al_2_O_3_/epoxy composites with varying filler contents (0–15 wt%) subjected to quasi-static tensile loading at strain rates of 0.001 s^−1^ and 0.01 s^−1^. Under both loading conditions, all specimens exhibited pronounced necking prior to final rupture. Further observation reveals that the macroscopic fracture modes display negligible strain-rate sensitivity: the fracture surfaces remain largely planar and are consistently oriented perpendicular to the applied tensile axis.

**Figure 9:**
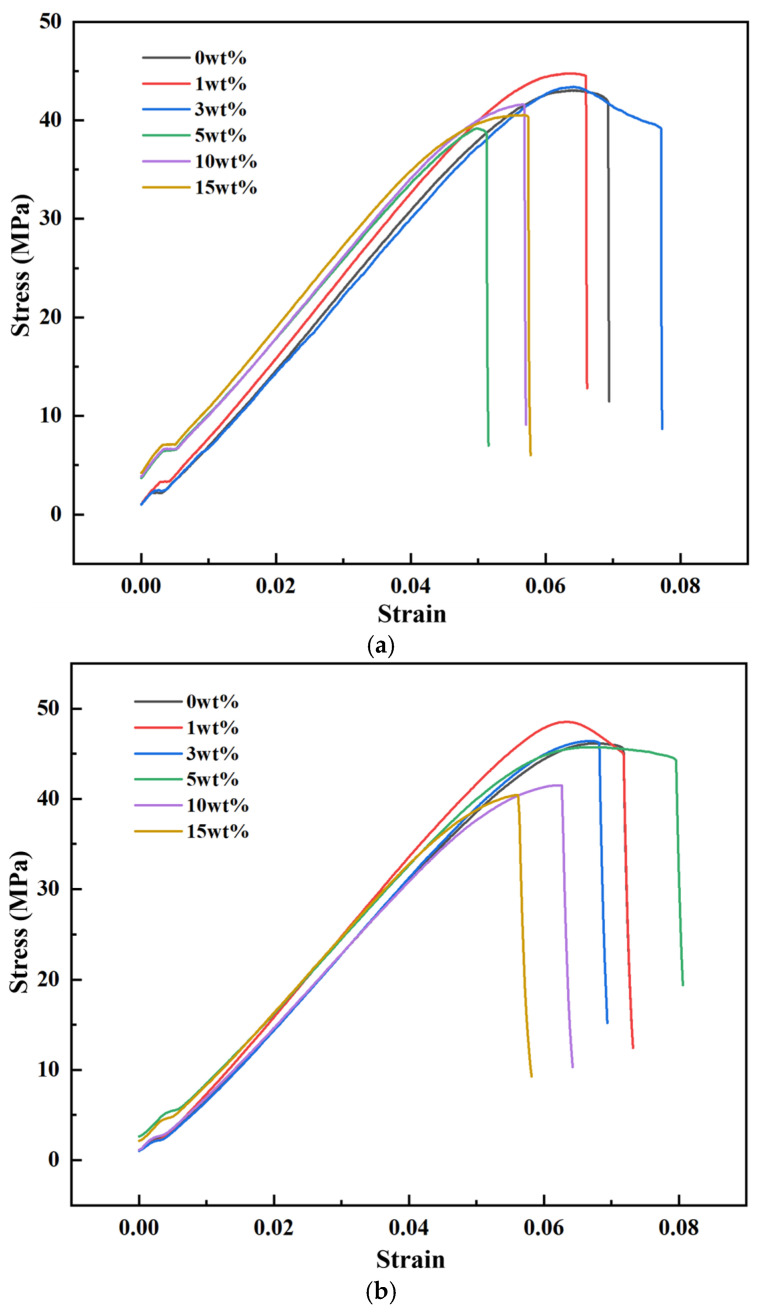
Stress–strain curves of nano-Al_2_O_3_/epoxy composites with varying nano-alumina contents at two strain rates: (**a**) Stress–strain curves at a strain rate of 0.001 s^−1^; (**b**) Stress–strain curves at a strain rate of 0.01 s^−1^.

Based on the quasi-static tensile test data, the stress–strain curves of the nano-Al_2_O_3_/epoxy composites at strain rates of 0.001 s^−1^ and 0.01 s^−1^ were plotted, as shown in Figure 9a and 9b, respectively. The tensile yield strengths of all specimens under these strain rates were extracted from the curves and are summarized in Table 3.

As evidenced by Figure 9a and Table 3, the composite with 1 wt% nano-Al_2_O_3_ content exhibits optimal tensile performance. As the filler content increases from 0 wt% to 1 wt%, the tensile strength, failure strain, and elastic modulus increase synchronously. Notably, the tensile strength of the 1 wt% specimen reaches the highest value among all groups, significantly surpassing that of the neat epoxy (0 wt%). However, with further increases in nano-Al_2_O_3_ content from 1 wt% to 3 wt%, 5 wt%, 10 wt%, and 15 wt%, the tensile properties gradually deteriorate. The tensile strength and failure strain of the 15 wt% specimen fall below those of the 1 wt% group, with certain metrics approaching or even dropping below those of the neat epoxy matrix.

Similarly, as illustrated in Figure 9b, the peak tensile stress rises as the nano-Al_2_O_3_ content increases from 0 wt% to 1 wt%, peaking at 1 wt%. Beyond this optimal point, a progressive decline in quasi-static tensile performance is observed with increasing filler content from 1 wt% to 3 wt%, 5 wt%, 10 wt%, and 15 wt%. The 15 wt% specimen exhibits a marked degradation in tensile properties, performing considerably worse than its 1 wt% counterpart.

Integrating the data presented in Figure 9 and Table 3 reveals that the neat epoxy resin exhibits a tensile strength of 43.04 MPa. Upon incorporation of 1 wt% nano-Al_2_O_3_, this value increases to 44.76 MPa. However, when the filler content is raised to 3 wt%, the tensile strength measures 43.40 MPa, which is statistically comparable to that of the neat matrix, indicating a significant attenuation of the reinforcement effect. At 5 wt%, the tensile strength drops markedly to 39.18 MPa, representing the lowest value among all groups; correspondingly, the peak stress in the stress–strain curves declines sharply, and the failure strain diminishes. This deterioration is primarily attributed to agglomeration induced by van der Waals interactions at elevated filler loadings, which generates stress concentration sites and disrupts the continuity of the matrix, thereby compromising both strength and ductility [66,67]. The same aggregation-driven degradation mechanism has been independently reported in other nano-Al_2_O_3_/epoxy systems: Duan et al. [67] explicitly attributed the reduction in fracture toughness at high filler contents to the presence of particle agglomerates in the epoxy matrix, and Zhang et al. [68] observed that even at substantial loadings (up to 18.4 wt%), the dominant fracture features remained void debonding, local plastic deformation, and crack pinning/deflection—mechanisms that are progressively suppressed as agglomeration increases and interfacial stress concentrations intensify. Therefore, the present finding that high loadings (≥3 wt%) fail to deliver monotonic reinforcement is not an artifact but a reproducible feature of nano-Al_2_O_3_/epoxy nanocomposites when the dispersion protocol is insufficient to overcome van der Waals re-agglomeration.

Further increasing the content to 10 wt% and 15 wt% exacerbates this degradation, with tensile strengths of 41.62 MPa and 40.54 MPa, respectively—both inferior to the neat epoxy. Consequently, the stress–strain curves manifest distinct characteristics of brittle fracture. These findings are consistent with the broader literature on nano-Al_2_O_3_/epoxy nanocomposites. For example, Yazman and Samancı [69] likewise observed an optimum at 1.0 wt% Al_2_O_3_, reporting that the tensile strength, Young’s modulus, and toughness of epoxy nanocomposites peaked at this loading (with ultimate tensile strength, modulus, and toughness increasing by 27.6%, 18.7%, and 187.9%, respectively, relative to the neat matrix) and deteriorated as the filler content was further increased to 1.25–2.0 wt%, a that 1 wt% is a representative optimum in Al_2_O_3_/epoxy systems. Similarly, Duan et al. [67] found that although 5 wt% 50 nm Al_2_O_3_ significantly enhanced the tensile modulus and strength of an epoxy resin, the quasi-static fracture toughness began to decline at high filler contents due to particle agglomeration, a trend that parallels the present observation of strength degradation beyond 1 wt% in this work. The present result—where a low loading (1 wt%) slightly enhances and a high loading (≥3 wt%) degrades tensile strength—therefore aligns with the consensus that nano-Al_2_O_3_ reinforcement in epoxy follows a non-monotonic dependence on filler content, with an optimal window located at very low loadings.

With the nano-Al_2_O_3_ content exceeding the optimal 1 wt%, the excess nanoparticles tend to agglomerate due to their high surface energy. This agglomeration fosters the formation of internal micropores and interfacial defects, undermining the structural integrity of the epoxy matrix. Acting as potent stress concentrators, these agglomerates induce severe localized stress elevations under external loading, serving as preferential sites for crack initiation and propagation. Concurrently, the compromised interfacial adhesion weakens the efficiency of load transfer from the matrix to the reinforcing particles, negating the intrinsic strengthening potential of the nano-Al_2_O_3_. Macroscopically, this synergistic effect manifests as a reduction in the peak tensile stress and failure strain, establishing a clear trend of mechanical degradation with increasing filler content.

In summary, the reinforcing effect of nano-Al_2_O_3_ on the epoxy matrix exhibits a distinct threshold, with 1 wt% identified as the optimal loading level that balances strength and ductility. Beyond this threshold, the reinforcement efficacy diminishes rapidly, and a significant degradation in mechanical performance is observed at higher filler loadings. Concurrently, the quasi-static tensile behavior demonstrates pronounced strain-rate sensitivity: the overall strength increases under the intermediate strain rate, while the plasticity of composites with higher filler contents exhibits a more intense response to strain rate variations.

**Figure 10:**
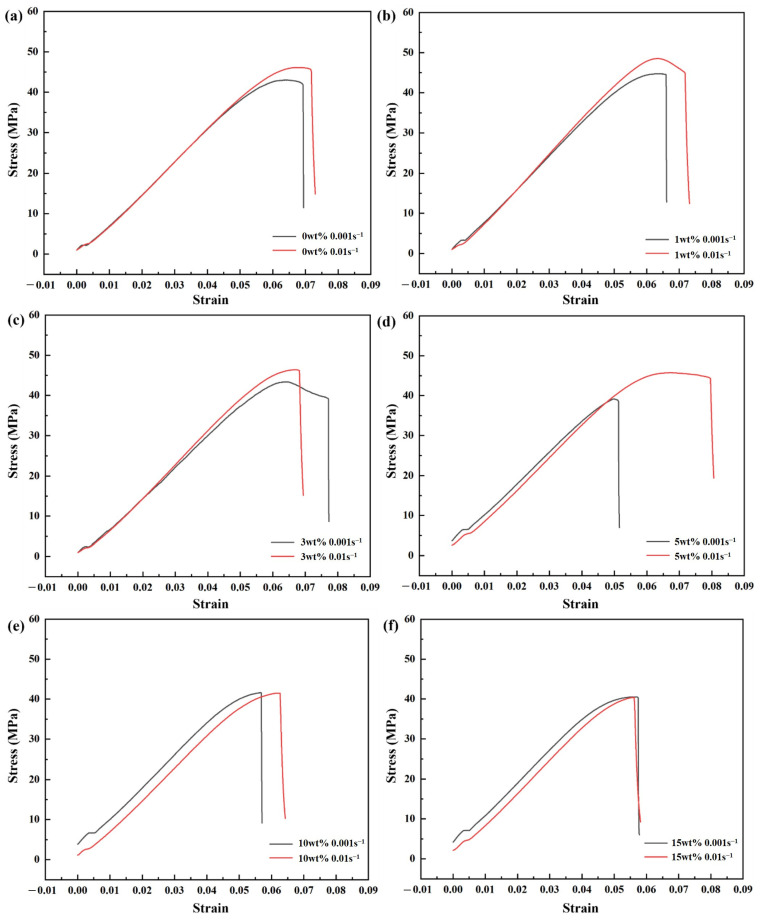
Stress–strain curves of nano-Al_2_O_3_/epoxy composites at different strain rates: (**a**) 0 wt% nano-Al_2_O_3_; (**b**) 1 wt% nano-Al_2_O_3_; (**c**) 3 wt% nano-Al_2_O_3_; (**d**) 5 wt% nano-Al_2_O_3_; (**e**) 10 wt% nano-Al_2_O_3_; (**f**) 15 wt% nano-Al_2_O_3_.

As evidenced by Figure 10 (stress–strain curves at two strain rates) and the tensile strength data in Table 3, the tensile properties of all compositions at the higher strain rate of 0.01 s^−1^ are superior to those at 0.001 s^−1^. Specifically, the tensile strength of the neat epoxy rises from 43.04 MPa to 46.12 MPa, representing an approximate increase of 7%. Similarly, the 1 wt% optimal composite and other formulations exhibit enhanced tensile strengths under the higher strain rate.

For composites with low filler contents (1 wt% and 3 wt%), the stress–strain curves at both strain rates display highly similar profiles during the elastic and yielding stages. While these materials exhibit positive strain-rate sensitivity, the fundamental characteristic of brittle fracture remains unchanged, and the failure strain remains relatively stable. In contrast, for composites with higher filler loadings (5 wt% and above), the low strain rate induces a steep fracture with poor plasticity. Under the high strain rate, however, a distinct yielding plateau or extended flow region emerges, accompanied by a significant elevation in failure strain. This divergence is attributed to the fact that interfacial defects stemming from agglomeration facilitate brittle fracture at low strain rates. Conversely, the high strain rate activates energy-dissipating mechanisms such as crazing and shear banding, which retard crack propagation and enhance toughness and ductility. Nevertheless, despite a marginal strength increase at 10 wt% and 15 wt% under high strain rates, their overall performance remains inferior to that of the low-filler-content composites and the neat matrix. This indicates that performance degradation dominated by particle agglomeration prevails, ultimately resulting in low-strength, low-ductility failure modes.

**Figure 11:**
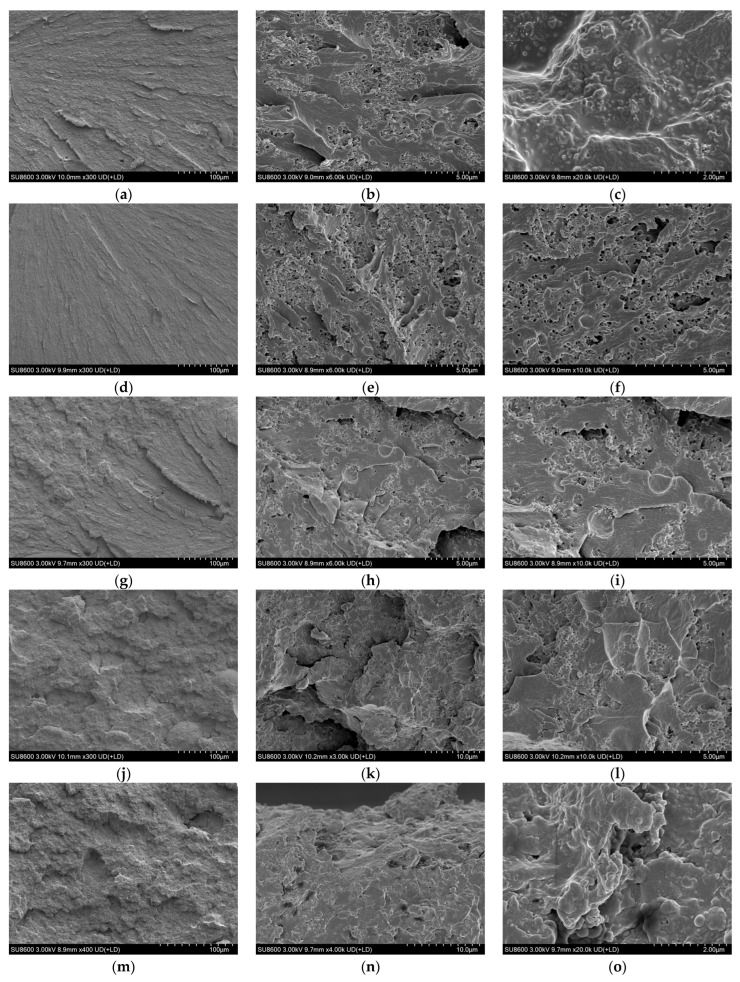

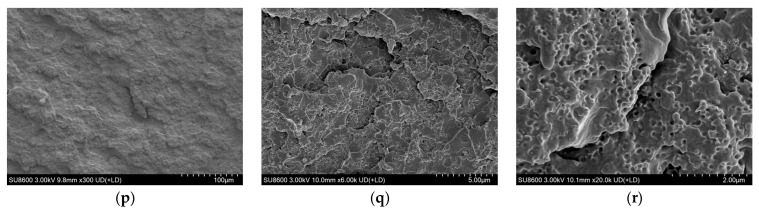
Microscopic fracture surface morphologies of nano-Al_2_O_3_/epoxy composite specimens with varying nano-Al_2_O_3_ contents after quasi-static tensile loading. (**a**) Fracture surface morphology of the quasi-static tensile specimen of neat epoxy resin at 300× magnification. (**b**) Fracture surface morphology of the quasi-static tensile specimen of neat epoxy resin at 6000× magnification. (**c**) Fracture surface morphology of the quasi-static tensile specimen of neat epoxy resin at 20,000× magnification. (**d**) Fracture surface morphology of the quasi-static tensile specimen of the 1 wt% nano-Al_2_O_3_/epoxy composite at 300× magnification. (**e**) Fracture surface morphology of the quasi-static tensile specimen of the 1 wt% nano-Al_2_O_3_/epoxy composite at 6000× magnification. (**f**) Fracture surface morphology of the quasi-static tensile specimen of the 1 wt% nano-Al_2_O_3_/epoxy composite at 10,000× magnification. (**g**) Fracture surface morphology of the quasi-static tensile specimen of the 3 wt% nano-Al_2_O_3_/epoxy composite at 300× magnification. (**h**) Fracture surface morphology of the quasi-static tensile specimen of the 3 wt% nano-Al_2_O_3_/epoxy composite at 6000× magnification. (**i**) Fracture surface morphology of the quasi-static tensile specimen of the 3 wt% nano-Al_2_O_3_/epoxy composite at 20,000× magnification. (**j**) Fracture surface morphology of the quasi-static tensile specimen of the 5 wt% nano-Al_2_O_3_/epoxy composite at 300× magnification. (**k**) Fracture surface morphology of the quasi-static tensile specimen of the 5 wt% nano-Al_2_O_3_/epoxy composite at 3000× magnification. (**l**) Fracture surface morphology of the quasi-static tensile specimen of the 5 wt% nano-Al_2_O_3_/epoxy composite at 10,000× magnification. (**m**) Fracture surface morphology of the quasi-static tensile specimen of the 10 wt% nano-Al_2_O_3_/epoxy composite at 400× magnification. (**n**) Fracture surface morphology of the quasi-static tensile specimen of the 10 wt% nano-Al_2_O_3_/epoxy composite at 4000× magnification. (**o**) Fracture surface morphology of the quasi-static tensile specimen of the 10 wt% nano-Al_2_O_3_/epoxy composite at 20,000× magnification. (**p**) Fracture surface morphology of the quasi-static tensile specimen of the 15 wt% nano-Al_2_O_3_/epoxy composite at 300× magnification. (**q**) Fracture surface morphology of the quasi-static tensile specimen of the 15 wt% nano-Al_2_O_3_/epoxy composite at 6000× magnification. (**r**) Fracture surface morphology of the quasi-static tensile specimen of the 15 wt% nano-Al_2_O_3_/epoxy composite at 20,000× magnification.

Following the quasi-static tensile tests, the fracture surfaces were examined using scanning electron microscopy (SEM). As shown in Figure 11a,b, two distinct morphologies are observed: one characterized by oblique shear slip bands and layered tear ridges distributed along the principal stress direction, indicating crack propagation along a single shear plane; the other featuring a multi-directionally disordered, rugged topography where cracks initiate at multiple defect sites and coalesce. Notably, neither morphology exhibits the mirror-like cleavage facets typical of brittle fracture, suggesting that shear deformation dominates the crack growth process. As illustrated in Figure 11c, the shear bands are populated with numerous nano- to micron-scale microvoids and cellular honeycomb-like pores. Furthermore, the uniformly distributed nanoparticulate phase-separated structures and entrapped curing-induced bubbles act as stress concentrators, facilitating crack initiation and propagation. This fracture mode confirms that the neat epoxy resin fails in a quasi-brittle manner under quasi-static tension.

Figure 11d,e present the fracture surfaces of the 1 wt% nano-Al_2_O_3_/epoxy composite. At low magnification (Figure 11d), the fracture surface appears highly rough, with a high density of oblique, layered tear ridges aligned with the principal stress axis. The roughness and ridge density exceed those of the neat epoxy, and the absence of a mirror zone indicates a significantly tortuous crack path. At higher magnifications (Figure 11e,f), the shear bands reveal extensive microvoids and honeycomb-like pores, accompanied by prominent crazing and drawn fibrous plastic deformation arising from shear yielding. Crucially, no significant particle agglomerates are detected, with only sporadic curing bubbles visible, indicating excellent dispersion of the 1 wt% nano-Al_2_O_3_ within the epoxy matrix. These observations demonstrate that the 1 wt% composite exhibits a shear-dominated fracture behavior, representing a transition from quasi-brittleness to ductility.

Figure 11g–l depict the fracture surfaces for the 3 wt% and 5 wt% nano-Al_2_O_3_ composites, respectively. As shown in Figure 11g,j at 300× magnification, both surfaces display a highly irregular and fragmented morphology. While the 3 wt% specimen retains some layered tear ridges, they are absent in the 5 wt% sample. Crack propagation becomes multi-directional and disorderly, following the interfaces of agglomerates, which typifies the fracture behavior dominated by agglomeration at higher loadings. High-magnification images reveal extensive cluster-like, macro-porous networks formed by particle agglomerates in both groups. Pronounced interfacial debonding between agglomerates and the matrix is evident, and the matrix plasticity is severely constrained, appearing only as debris fragments within the inter-agglomerate spaces. This further corroborates that agglomeration and interfacial debonding constitute the primary fracture mechanisms.

Finally, Figure 11m–r correspond to the 10 wt% and 15 wt% nano-Al_2_O_3_ composites, respectively. At low magnification (Figure 11m,p), the fracture surfaces exhibit an extremely rough, particle-packed morphology devoid of any layered tearing features. Extensive micron-scale porous networks, resulting from severe particle agglomeration, permeate the surface, completely compromising the structural integrity of the epoxy matrix. As revealed at higher magnifications (Figure 11n at 4000× and Figure 11q at 6000×), distinct micron-sized Al_2_O_3_ agglomerates are visible. Catastrophic interfacial debonding has occurred, leaving behind large voids. The epoxy matrix is reduced to fine debris confined to the interstices of the agglomerates, with cracks propagating randomly along the agglomerate interfaces. There is no evidence of toughening mechanisms such as particle bridging or crack deflection. These results indicate a total loss of dispersion efficacy at 10 wt% and 15 wt% loadings. Interface flaws induced by severe agglomeration become the dominant failure mechanism, nullifying any potential particle-toughening effects and resulting in classical brittle fracture with significantly degraded mechanical properties. Further high-magnification analysis (Figure 11o,r at 20,000×) reaffirms the presence of micron-scale agglomerates and pervasive interfacial voids, confirming that the material’s failure is governed by agglomerate-driven brittleness rather than particulate reinforcement.

In summary, at a nano-Al_2_O_3_ loading of 1 wt%, the nanoparticles are uniformly dispersed, and the fracture mode transitions from quasi-brittle to a quasi-brittle/ductile mixed mode, yielding optimal mechanical performance. The 3 wt% loading represents the critical agglomeration threshold, where intensified particle clustering diminishes the toughening efficacy and shifts the fracture behavior back toward brittleness. At 5 wt%, an inflection point for degradation is reached; the formation of continuous defect networks via agglomeration nullifies the nano-reinforcement mechanism, resulting in outright brittle fracture. Loadings of 10 wt% and 15 wt% constitute excessive additions, wherein the nanoparticles form continuous agglomerated structures that crush and displace the epoxy matrix. This leads to complete brittle fracture characterized by interfacial separation, accompanied by a significant deterioration in mechanical properties.

### 3.2. Analysis of Dynamic Tensile Test Results

**Figure 12:**
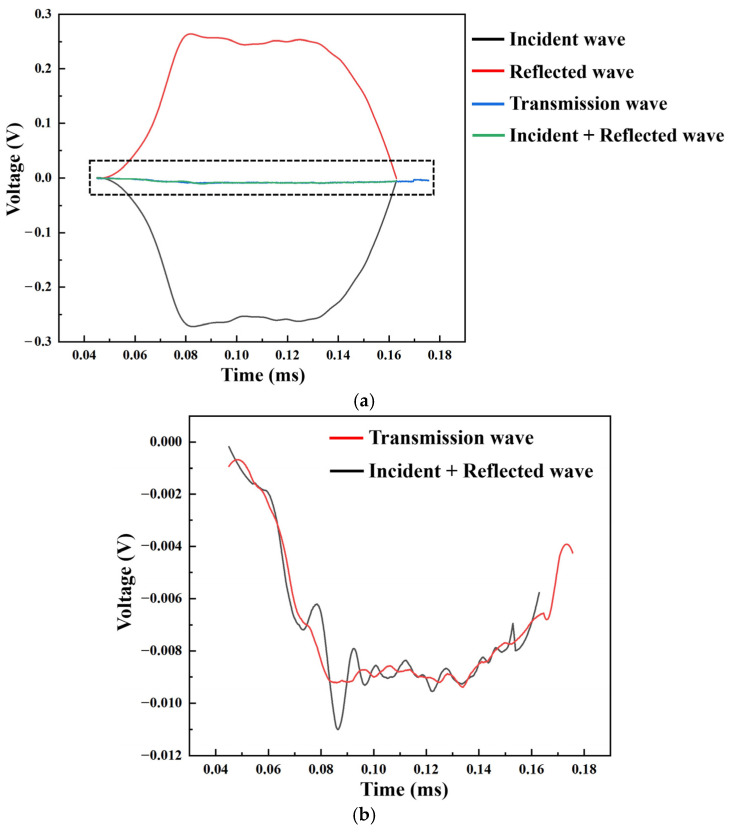
Three-wave verification waveforms from the SHPB test: (**a**) Raw voltage signals of the incident, reflected, and transmitted waves within the range of –0.3 V to 0.3 V; (**b**) Enlarged view highlighting the superposition of the incident plus reflected waves ($\varepsilon_{i}+\varepsilon_{r}$) and the transmitted wave ($\varepsilon_{t}$).

In SHTB data processing, a consistency check of the incident, reflected, and transmitted wave signals is essential. The stress equilibrium at the specimen interfaces must be verified based on one-dimensional stress wave theory, ensuring that the specimen undergoes uniform deformation during the test. This condition is expressed as follows:

(4) $$\varepsilon_{i}+\varepsilon_{r}=\varepsilon_{t}$$

where $\varepsilon_{i}$, $\varepsilon_{r}$ and $\varepsilon_{t}$ denote the incident, reflected, and transmitted strains, respectively. Figure 12a,b illustrate the three-wave verification profiles. Given that the epoxy composites examined herein possess a low acoustic impedance relative to the pressure bars, a significant portion of the incident stress wave is reflected at the bar/specimen interfaces (Figure 12b). Consequently, the amplitude of the transmitted wave ($\varepsilon_{t}$, blue curve) is substantially lower than those of the incident and reflected waves. Despite this apparent disparity in magnitude, careful inspection reveals that the superimposed signal ($\varepsilon_{i}+\varepsilon_{r}$, green curve) closely matches the transmitted wave ($\varepsilon_{t}$) in both waveform trend and phase. Both signals oscillate marginally around zero with negligible DC drift, indicating minimal waveform dispersion or distortion during propagation. Furthermore, the satisfaction of the $\varepsilon_{i}+\varepsilon_{r}\approx \varepsilon_{t}$ condition confirms the establishment of stress equilibrium. These observations collectively validate the proper alignment of the experimental setup and the reliability of the acquired dynamic response data.

**Figure 13:**
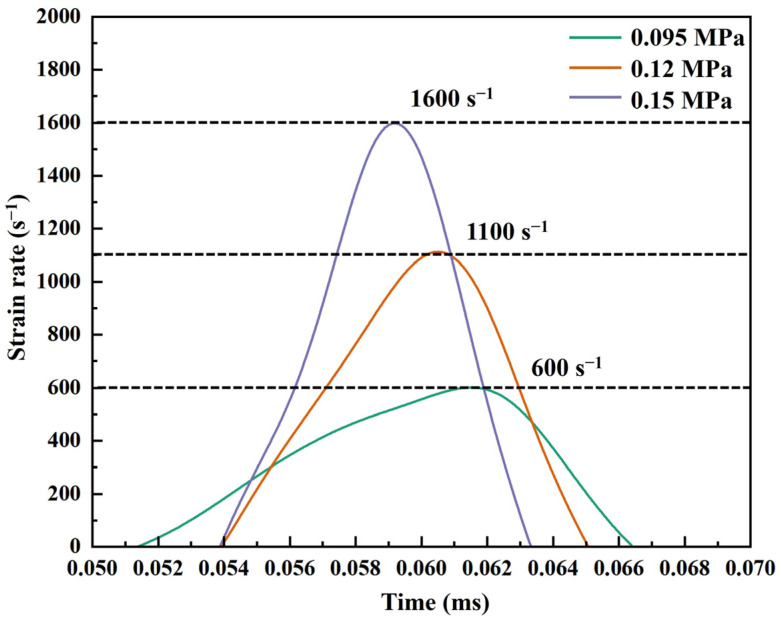
Strain rate–time curves for specimens tested at loading pressures of 0.095, 0.12, and 0.15 MPa.

Figure 13 presents the strain rate–time curves obtained under loading pressures of 0.05 MPa, 0.12 MPa, and 0.15 MPa. The corresponding average strain rates for these three firing pressures are approximately 600 s^−1^, 1100 s^−1^, and 1600 s^−1^, respectively.

**Figure 14:**
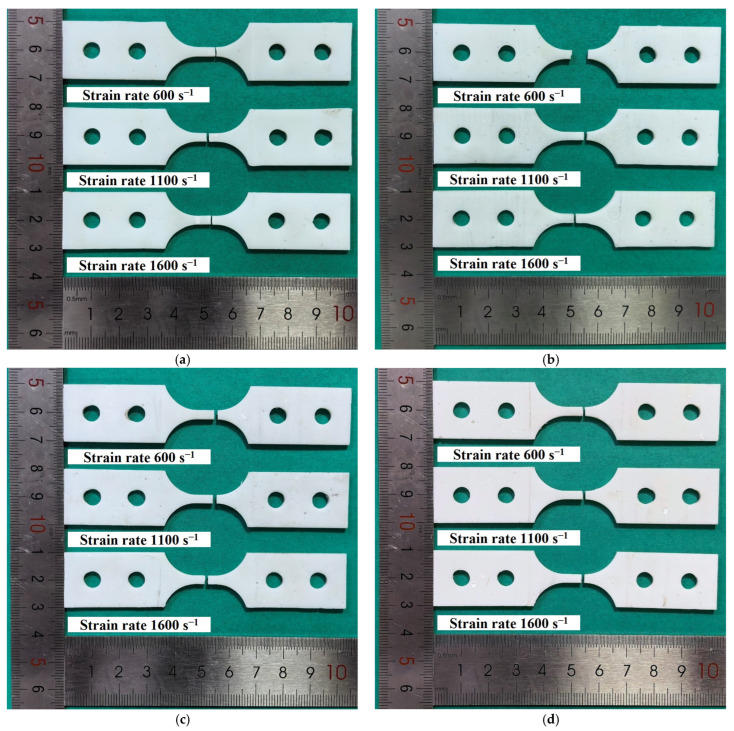
Macroscopic fracture surface morphologies of nano-Al_2_O_3_/epoxy composite specimens subjected to dynamic tensile loading at varying strain rates. (**a**) Fracture surface morphology of the 0 wt% nano-Al_2_O_3_/epoxy composite specimen at different strain rates. (**b**) Fracture surface morphology of the 5 wt% nano-Al_2_O_3_/epoxy composite specimen at different strain rates. (**c**) Fracture surface morphology of the 10 wt% nano-Al_2_O_3_/epoxy composite specimen at different strain rates. (**d**) Fracture surface morphology of the 15 wt% nano-Al_2_O_3_/epoxy composite specimen at different strain rates.

Figure 14 presents the dynamic tensile fracture morphologies of nano-Al_2_O_3_/epoxy composite specimens with nano-Al_2_O_3_ loadings of 0 wt%, 5 wt%, 10 wt%, and 15 wt%, acquired under strain rates of 600 s^−1^ (corresponding drive gas pressure: 0.095 MPa), 1100 s^−1^ (0.12 MPa), and 1600 s^−1^ (0.15 MPa). As evidenced by the figure, all specimens fractured exclusively within the gauge section, with fracture surfaces oriented strictly perpendicular to the tensile loading direction, collectively exhibiting predominant quasi-brittle fracture characteristics.

**Figure 15:**
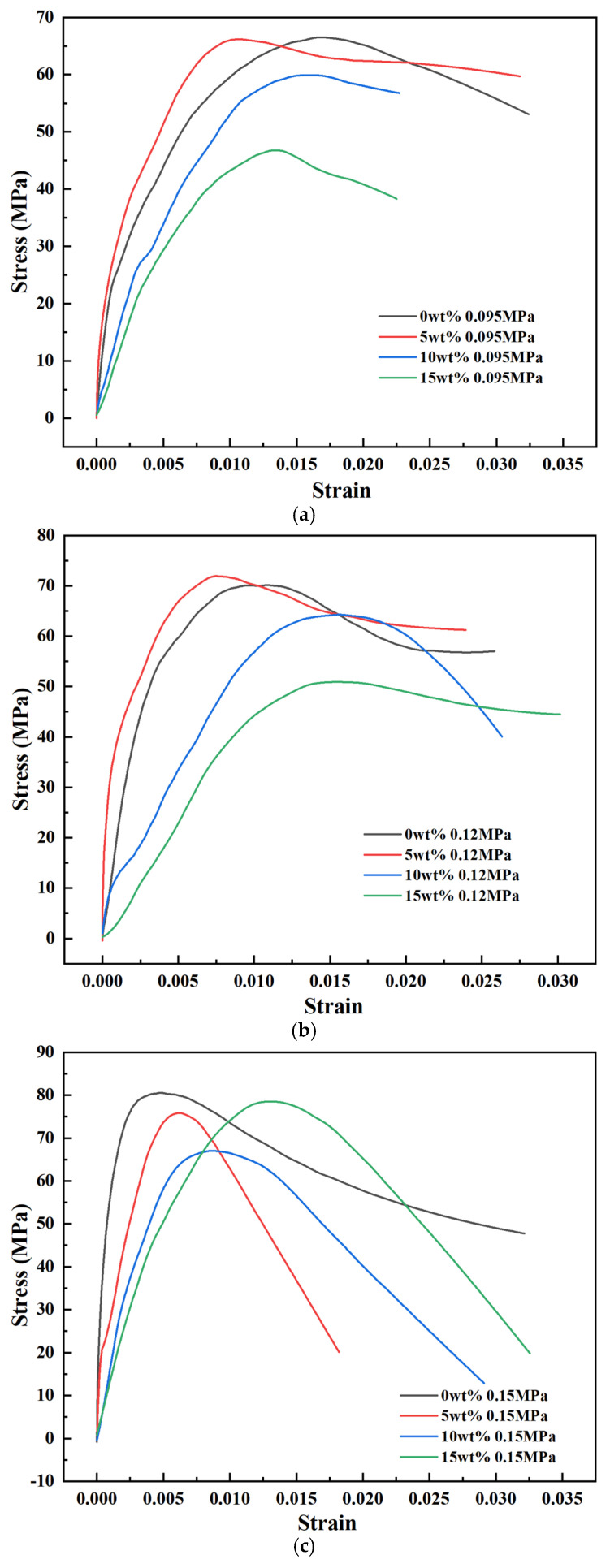
Dynamic tensile stress–strain curves of nano-Al_2_O_3_/epoxy composite specimens with varying nano-Al_2_O_3_ loadings (0–15 wt%) tested under varying drive gas pressures: (**a**) Dynamic tensile stress–strain curves under a drive gas pressure of 0.095 MPa; (**b**) Dynamic tensile stress–strain curves under a drive gas pressure of 0.12 MPa; (**c**) Dynamic tensile stress–strain curves under a drive gas pressure of 0.15 MPa.

Figure 15a presents the dynamic tensile stress–strain curves of the four nano-Al_2_O_3_/epoxy composite groups with nano-Al_2_O_3_ loadings of 0, 5, 10, and 15 wt%, tested under a drive gas pressure of 0.095 MPa. All curves exhibit a characteristic three-stage evolution: near-linear elastic ascent, yielding to peak stress, and subsequent strain softening. The initial segments show near-linear, steep stress increases corresponding to the elastic deformation regime, with stress rising uniformly with strain and no discernible pre-yield plastic deformation. Beyond the elastic regime, the curves progressively flatten as they enter the plastic yielding stage: the stress accumulation rate decelerates continuously until each specimen reaches its respective peak stress, followed by a slow post-peak stress decline with increasing strain until final fracture. This response collectively reflects the canonical dynamic tensile mechanical behavior of polymer resin–matrix composites. Extraction of peak stress-associated strains reveals that the characteristic strain at peak stress shifts progressively to higher values with increasing nano-Al_2_O_3_ loading. Concurrently, the slope of the initial elastic segment—a direct proxy for the elastic modulus—decreases monotonically with higher nano-Al_2_O_3_ content, indicating that nano-Al_2_O_3_ incorporation partially compromises the tensile strength of the epoxy matrix under the 0.095 MPa drive gas pressure.

Figure 15b plots the dynamic tensile stress–strain curves of the same composite series tested under a drive gas pressure of 0.12 MPa. Consistent with the 0.095 MPa case, the curves follow the identical three-stage evolution. All specimens exhibit near-linear stress growth in the initial elastic regime: the neat epoxy (0 wt%) and 5 wt% nano-Al_2_O_3_/epoxy specimens show steeper initial slopes, corresponding to superior elastic moduli. The initial slope decreases progressively with increasing nano-Al_2_O_3_ loading, with the 15 wt% high-loading specimen exhibiting the most gradual elastic ascent, reflecting a diminished initial resistance to deformation. Upon entering the plastic deformation stage, the stress accumulation rate decelerates continuously across all specimens, each reaching its respective peak stress at distinct strain levels. Notably, the 5 wt% composite attains its peak stress earliest, associated with the smallest strain interval to peak; conversely, the peak strains for the 10 wt% and 15 wt% specimens shift progressively to higher values. Furthermore, the magnitude and rate of post-peak stress decay increase markedly with higher nano-Al_2_O_3_ content, confirming that nano-Al_2_O_3_ incorporation similarly degrades the tensile strength of the epoxy matrix under the 0.12 MPa drive gas pressure.

Figure 15c displays the dynamic tensile stress–strain curves of the nano-Al_2_O_3_/epoxy composite specimens tested under a drive gas pressure of 0.15 MPa. While the characteristic three-stage evolution (elastic ascent, peak stress, strain softening) persists across all four composite groups, the overall peak stress levels are uniformly elevated compared to the lower-pressure conditions (0.095 MPa and 0.12 MPa). The neat epoxy (0 wt%) exhibits the steepest initial slope among the four groups, corresponding to the highest initial elastic modulus; the slope of the elastic ascent segment decreases sequentially with increasing nano-Al_2_O_3_ loading, reflecting a progressive reduction in the initial resistance to deformation. In the plastic deformation stage, the neat epoxy attains its peak stress first, followed by the 5 wt%, 10 wt%, and 15 wt% composites, whose peak strains shift progressively to higher values in turn. Post-peak stress decays rapidly and continuously with further increasing strain until final fracture, further confirming that nano-Al_2_O_3_ incorporation degrades the tensile strength of the epoxy matrix even under the 0.15 MPa drive gas pressure.

**Figure 16:**
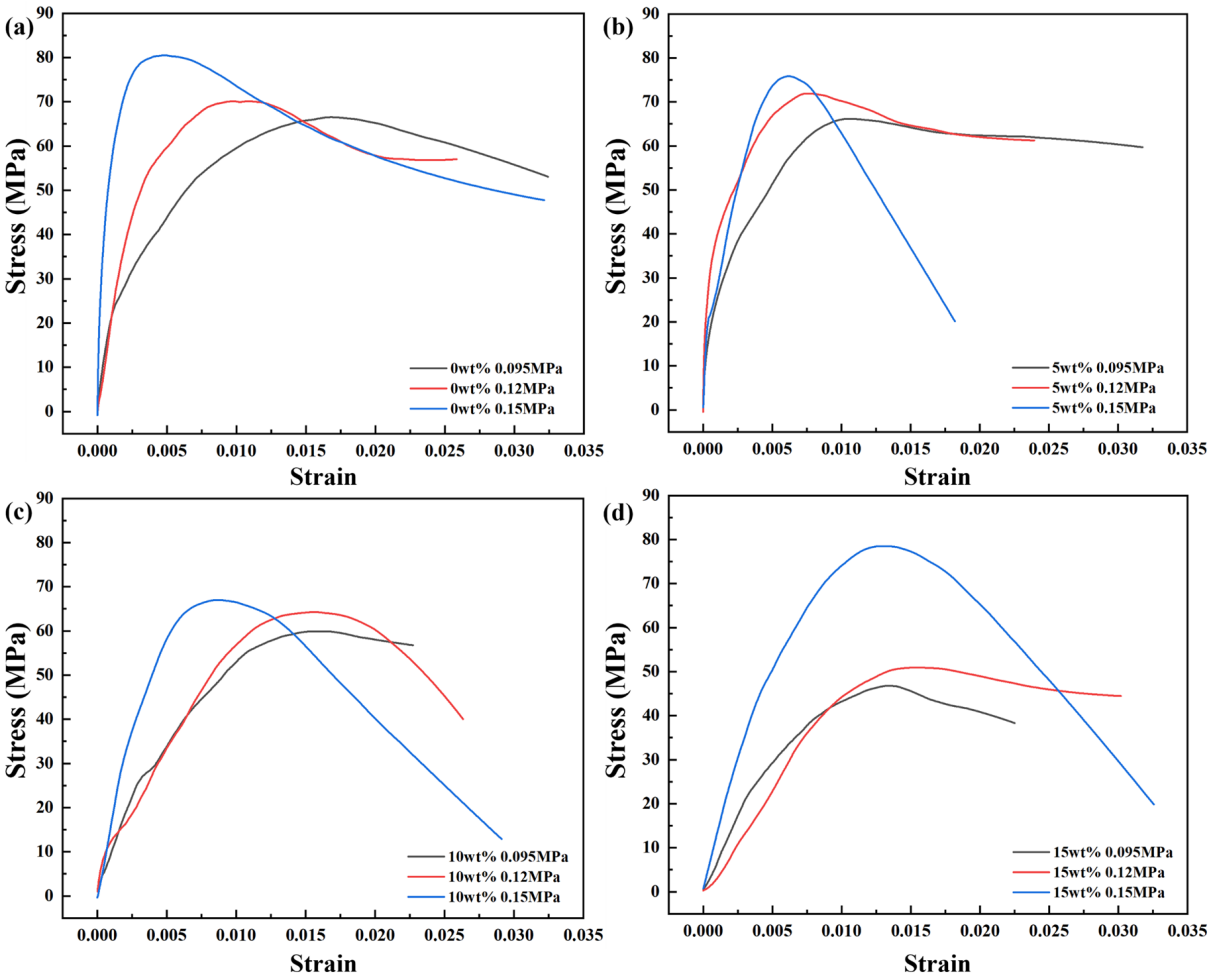
Dynamic tensile stress–strain curves of nano-Al_2_O_3_/epoxy composites under varying drive gas pressures: (**a**) 0 wt% nano-Al_2_O_3_; (**b**) 5 wt% nano-Al_2_O_3_; (**c**) 10 wt% nano-Al_2_O_3_; (**d**) 15 wt% nano-Al_2_O_3_.

Figure 16 presents the dynamic tensile stress–strain curves of nano-Al_2_O_3_/epoxy composites with varying nano-Al_2_O_3_ loadings tested under different drive gas pressures. As the strain rate increases progressively from 600 s^−1^ (corresponding to a drive gas pressure of 0.095 MPa) to 1100 s^−1^ (0.12 MPa) and further to 1600 s^−1^ (0.15 MPa), the peak stress (i.e., tensile strength) of all composite groups rises markedly, accompanied by significantly enhanced ultimate load-bearing capacity. Concurrently, the slope of the initial elastic segment of the stress–strain curves increases monotonically with strain rate, corresponding to an elevated dynamic elastic modulus—this indicates that the epoxy matrix exhibits substantially improved initial resistance to deformation under high-rate loading. Furthermore, elevated strain rates modify the deformation evolution characteristics of the composites: the characteristic strain at peak stress decreases progressively at higher strain rates, signifying accelerated onset of yielding and damage. Collectively, these observations demonstrate a pronounced positive strain-rate sensitivity of the nano-Al_2_O_3_/epoxy composites.

Table 4 presents the tensile strengths of nano-Al_2_O_3_/epoxy composites with varying nano-Al_2_O_3_ loadings tested under different drive gas pressures. As the drive gas pressure increases sequentially from 0.095 MPa to 0.12 MPa and further to 0.15 MPa (corresponding to a progressive elevation in the dynamic tensile strain rate), the tensile strength of all composite groups exhibits a consistent upward trajectory. Specifically, the neat epoxy (0 wt%) records tensile strengths of 66.52 MPa, 70.11 MPa, and 80.54 MPa across the three pressure levels; the 5 wt% nano-Al_2_O_3_/epoxy composite yields values of 59.91 MPa, 64.30 MPa, and 67.02 MPa; the 10 wt% group reaches 66.14 MPa, 71.92 MPa, and 75.85 MPa; while the 15 wt% composite shows measurements of 46.77 MPa, 50.92 MPa, and 78.49 MPa. Collectively, all four material systems demonstrate a synchronous enhancement in tensile strength with increasing strain rate, with the magnitude of strength gain in the high-pressure interval (0.12–0.15 MPa) markedly exceeding that in the low-pressure interval (0.095–0.12 MPa). This trend underscores that the load-bearing capacity of the composites exhibits accelerated growth with rising loading rates, a characteristic manifestation of positive strain-rate sensitivity in thermosetting polymer matrices.

Collectively, based on the dynamic tensile tests of nano-Al_2_O_3_/epoxy composites with varying nano-Al_2_O_3_ loadings conducted under different drive gas pressures—in conjunction with the stress–strain curves and tensile strength data presented in Figure 15 and Figure 16, and Table 4—all composite formulations exhibit a pronounced positive strain-rate sensitivity: the tensile strength of every group increases monotonically with rising drive gas pressure and the corresponding dynamic strain rate. This positive strain-rate sensitivity is consistent with the established behavior of nano-Al_2_O_3_-modified epoxy under dynamic loading. Duan et al. [67] investigated the dynamic fracture behavior of nano-Al_2_O_3_/epoxy using a SHPB combined with two-dimensional digital image correlation, and demonstrated that crack propagation velocity can be reduced and dynamic initiation fracture toughness can be increased by nano-Al_2_O_3_ addition, while noting that the particle-size effect on dynamic fracture toughness becomes weaker once the filler content enters the high-loading regime where agglomeration dominates. This mirrors the present observation that the 15 wt% composite, despite its severe strength attenuation at low-to-intermediate rates, recovers to 78.49 MPa at 1600 s^−1^—approaching the neat epoxy’s 80.54 MPa—indicating that at sufficiently high strain rates, energy-dissipation mechanisms such as crack pinning and localized plastic tearing can partially offset the detrimental effect of agglomeration. It should be noted, however, that direct comparisons of absolute dynamic tensile strength values across different studies remain difficult, because the available high-rate data for nano-Al_2_O_3_/epoxy systems are predominantly obtained under dynamic fracture or dynamic compressive loading [67], and standardized SHTB tensile databases at 600–1600 s^−1^ for this specific material system are still limited. The present work contributes to closing this gap by providing a systematic dynamic tensile dataset for 0, 5, 10, and 15 wt% nano-Al_2_O_3_/epoxy over a well-controlled strain-rate range.

Overall, the incorporation of nano-Al_2_O_3_ generally reduces the dynamic tensile strength of the epoxy matrix. Only the moderate 5 wt% loading marginally retains a load-bearing capacity comparable to that of the neat epoxy under low-to-intermediate strain rates. In contrast, the high loadings of 10 wt% and 15 wt% induce marked strength degradation, with the 15 wt% excessive loading causing the most severe attenuation of tensile performance.

The underlying mechanism for this strength reduction lies in the combined effects of two factors: (i) interfacial defects and particle agglomeration induced by nano-Al_2_O_3_ incorporation, and (ii) the intrinsic viscoelastic, strain-rate-dependent mechanical behavior of the epoxy matrix itself. These coupled effects govern the load-bearing capacity and deformation-damage evolution of the composites. Macroscopically, the mechanical response of the system is dominated by the inherent viscoelasticity of the epoxy matrix; meanwhile, the internal microstructural defects arising from nano-Al_2_O_3_ addition modulate the composite’s load-bearing capacity and damage evolution kinetics in a strain-rate-dependent manner, jointly dictating the overall failure behavior across all tested loading conditions.

Post-dynamic tensile testing, the fracture surfaces of the neat epoxy resin and nano-Al_2_O_3_/epoxy composites with varying nano-Al_2_O_3_ loadings were characterized via SEM.

**Figure 17:**
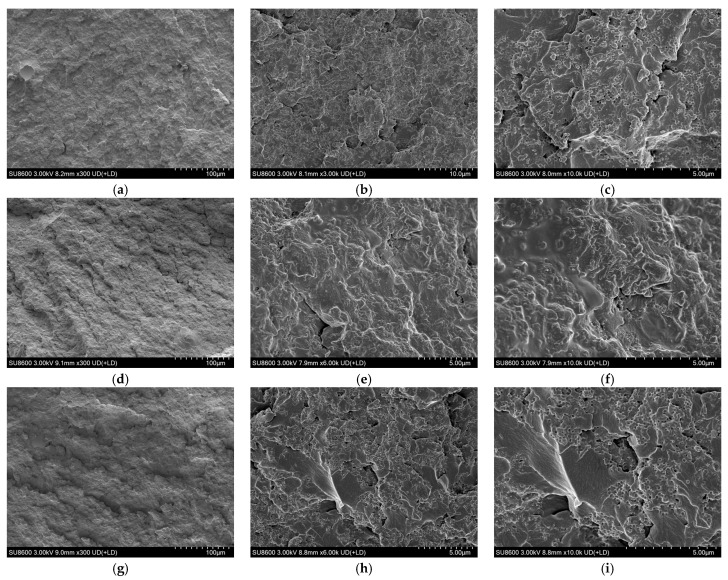
Microscopic fracture surface morphologies of nano-Al_2_O_3_/epoxy composite specimens with varying nano-Al_2_O_3_ loadings after dynamic tensile loading. (**a**) Fracture surface morphology of the dynamic tensile specimen of neat epoxy resin at 300× magnification. (**b**) Fracture surface morphology of the dynamic tensile specimen of neat epoxy resin at 3000× magnification. (**c**) Fracture surface morphology of the dynamic tensile specimen of neat epoxy resin at 10,000× magnification. (**d**) Fracture surface morphology of the dynamic tensile specimen of the 5 wt% nano-Al_2_O_3_/epoxy composite at 300× magnification. Fracture surface morphology of the dynamic tensile specimen of the 5 wt% nano-Al_2_O_3_/epoxy composite at 6000× magnification. (**e**) Fracture surface morphology of the dynamic tensile specimen of the 5 wt% nano-Al_2_O_3_/epoxy composite at 6000× magnification. (**f**) Fracture surface morphology of the dynamic tensile specimen of the 5 wt% nano-Al_2_O_3_/epoxy composite at 10,000× magnification. (**g**) Fracture surface morphology of the dynamic tensile specimen of the 10 wt% nano-Al_2_O_3_/epoxy composite at 300× magnification. (**h**) Fracture surface morphology of the dynamic tensile specimen of the 10 wt% nano-Al_2_O_3_/epoxy composite at 6000× magnification. (**i**) Fracture surface morphology of the dynamic tensile specimen of the 10 wt% nano-Al_2_O_3_/epoxy composite at 10,000× magnification.

Figure 17a–c present the fracture surface morphologies of the neat epoxy dynamic tensile specimens. As shown in Figure 17a at 300× magnification, the fracture surface exhibits an overall rough, irregular topography devoid of discernible plastic flow traces or oriented fibrous stretching structures. No large-area, flat cleavage facets are observed, and the fracture surface displays a fully disseminated, non-directional rough fracture feature. At higher magnification (3000×, Figure 17b), abundant irregular fracture steps, tear ridges, and discretely distributed microvoids and dimples are resolved on the fracture surface. The tear ridges propagate in a disordered, non-unidirectional manner, with sharp, well-defined edges bordering the fracture steps. Further magnified to 10,000× (Figure 17c), no evidence of matrix plastic stretching is detectable. The microvoids primarily nucleate via the coalescence of microcracks during brittle fracture, and the undulating topographic features of the fracture surface consist entirely of fracture steps and tear ridges generated by the intersection of discrete fracture planes. Collectively, the microscopic morphology of the neat epoxy dynamic tensile fracture surface exhibits classic brittle fracture characteristics.

Figure 17d–f display the fracture surface morphologies of the 5 wt% nano-Al_2_O_3_/epoxy dynamic tensile specimen. As illustrated in Figure 17d at 300× magnification, the fracture surface is highly rough with pronounced topographic relief, free of large-area flat brittle cleavage facets or the single dominant crack propagation path commonly observed in neat epoxy fractures. At 6000× magnification (Figure 17e), the fracture surface features abundant irregular tear ridges and fracture steps, with individual nano-Al_2_O_3_ particles clearly resolved. Microwoids and crazing propagation traces are visible surrounding the embedded particles. Critically, the crack path becomes significantly more tortuous due to crack pinning and deflection by nanoparticles: the rapid brittle crack propagation typical of the neat epoxy is induced to bifurcate, forming a complex interconnected fracture network. Further magnified to 10,000× (Figure 17f), the nano-Al_2_O_3_ particles exhibit robust interfacial bonding with the epoxy matrix, with no discernible interfacial debonding or pull-out. Locally, wrinkled torn matrix morphologies—resulting from tensile stretching around embedded particles—are observed. The fracture surface roughness is markedly elevated, with tear ridges distributed at higher density; the flat step features characteristic of brittle fracture are attenuated, giving way to a hybrid fracture morphology dominated by nanoparticle-induced toughening effects, with notably weakened brittle fracture signatures.

Figure 17g–i present the fracture surface morphologies of the 10 wt% nano-Al_2_O_3_/epoxy dynamic tensile specimen. As shown in Figure 17g at 300× magnification, the fracture surface exhibits pronounced rough relief without flat brittle cleavage facets, displaying a disseminated fracture feature. At higher magnifications (Figure 17h,i), the fracture surface is populated with abundant nanoparticle-induced microvoids, crazes, and tear ridges. The crack path is rendered tortuous by crack pinning and deflection at particle interfaces, with robust interfacial adhesion and no observable debonding. The fracture morphology has transitioned from the brittle step-like topography of the neat epoxy to a hybrid configuration dominated by microvoids, particle-induced crazing, and localized matrix plastic tearing. Brittle fracture characteristics are markedly attenuated, demonstrating the notable enhancement of dynamic fracture toughness imparted by nano-Al_2_O_3_ incorporation.

**Figure 18:**
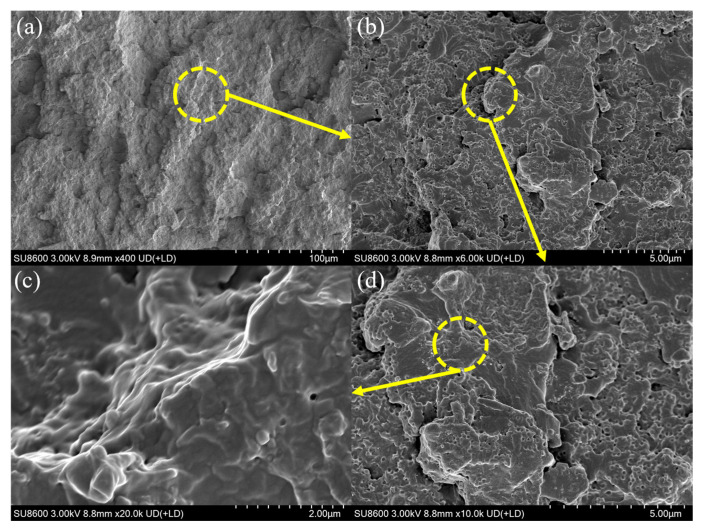
Fracture surface morphologies of the 15 wt% nano-Al_2_O_3_/epoxy composite specimen after dynamic tensile loading: (**a**) 400×; (**b**) 6000×; (**c**) 20,000×; (**d**) 10,000×.

Figure 18 presents the fracture surface morphologies of the dynamic tensile specimen of the 15 wt% nano-Al_2_O_3_/epoxy composite. As shown in Figure 18a at 400× magnification, the fracture surface exhibits pronounced topographic relief, devoid of flat brittle cleavage facets, and displays characteristic disseminated fracture features. At higher magnifications—Figure 18b (6000×) and Figure 18d (10,000×)—discretely distributed nano-Al_2_O_3_ particles are resolved across the fracture surface, surrounded by microvoids and propagation traces of crazes, a phenomenon induced by stress concentration around nanoparticles under dynamic loading. The embedded particles trigger massive microdamage initiation and propagation within the matrix: tear ridges are densely distributed with tortuous alignment, and subtle plastic wrinkles resulting from tensile stretching of the matrix are visible along ridge edges. At 20,000× magnification (Figure 18c), the nano-Al_2_O_3_ particles exhibit robust interfacial adhesion with the epoxy matrix, with no discernible interfacial debonding or pull-out; tensile flow traces and peripheral microvoids are clearly observed around the embedded particles. Collectively, the fracture morphology has transitioned from the brittle step-like topography characteristic of the neat epoxy to a hybrid configuration dominated by particle-induced microvoids, craze propagation, and localized matrix plastic tearing. Brittle fracture characteristics are markedly attenuated, demonstrating the effective enhancement of dynamic fracture toughness imparted by nano-Al_2_O_3_ incorporation into the epoxy matrix.

As synthesized from the dynamic tensile test results presented in Figure 14, Figure 15 and Figure 16, all recorded stress–strain curves follow a characteristic three-stage evolution: near-linear elastic ascent, yielding to peak stress, and subsequent strain softening. Elevated strain rates enhance both the peak stress and the initial elastic modulus of the curves, whereas increasing nano-Al_2_O_3_ loading monotonically reduces the slope of the initial elastic segment and the overall peak stress. Collectively, all specimens exhibit pronounced positive strain-rate sensitivity, whereby tensile strength improves systematically with rising strain rate. The incorporation of nano-Al_2_O_3_ generally degrades the tensile performance of the epoxy matrix: only the moderate 5 wt% loading retains a load-bearing capacity comparable to that of the neat epoxy, while the 15 wt% excessive loading induces the most severe strength attenuation. Macroscopically, all specimens fracture exclusively within the gauge section in a quasi-brittle manner. Microscopically, the neat epoxy displays a classic brittle fracture morphology; with increasing nano-Al_2_O_3_ content, the fracture surface transitions to a hybrid configuration with markedly attenuated brittle characteristics and enhanced toughness, supported by robust interfacial adhesion between the nano-Al_2_O_3_ particles and the epoxy matrix. This microstructural transition is in good agreement with the fracture-surface observations reported for other nano-Al_2_O_3_/epoxy systems. Zhang et al. [68] systematically examined the fracture surfaces of tensile and compact-tension specimens of Al_2_O_3_-filled E-54/DDS epoxy using SEM and AFM, and identified cavity/debonding of nanofiller, local plastic deformation, and crack pinning/deflection as the operative toughening mechanisms in the presence of nano-Al_2_O_3_. Duan et al. [67] similarly reported that dynamic fracture surfaces of nano-Al_2_O_3_/epoxy show rougher morphology and reduced crack velocity relative to the neat matrix. The present SEM observations—namely, the gradual disappearance of flat cleavage facets, the emergence of microvoids, tear ridges, and craze traces, and the retention of strong particle–matrix interfacial bonding without pull-out—thus corroborate the micro-mechanistic picture established in the prior literature, while extending it to higher filler loadings (10 and 15 wt%) where the hybridization of fracture modes is most pronounced.

## 4. Conclusions

This study presents a systematic investigation into the quasi-static and dynamic tensile behaviors of nano-Al_2_O_3_-reinforced epoxy composites across a wide filler loading range (0–15 wt%), combining macro-mechanical testing, high-strain-rate experimentation, and microscopic fracture analysis to clarify the content-dependent regulation mechanism of nano-Al_2_O_3_ on composite performance. The core conclusions are summarized as follows:

(1) For quasi-static tensile performance, 1 wt% is identified as the optimal nano-Al_2_O_3_ loading that balances tensile strength and ductility, achieving a peak strength of 44.76 MPa at 0.001 s^−1^, which is 4.0% higher than that of the neat epoxy. A critical agglomeration threshold emerges at 3 wt%, where intensified particle clustering begins to degrade mechanical performance. At 5 wt%, the composite reaches an inflection point of performance degradation, as continuous defect networks formed by agglomerates nullify the nano-reinforcement effect, leading to overt brittle fracture. Loadings of 10 wt% and 15 wt% represent excessive additions: severe agglomeration crushes the epoxy matrix, resulting in complete brittle failure dominated by interfacial separation and a significant reduction in both strength and plasticity.

(2) For dynamic tensile performance under strain rates of 600–1600 s^−1^, all composites exhibit pronounced positive strain-rate sensitivity, with peak stress and elastic modulus increasing monotonically with strain rate. Nano-Al_2_O_3_ incorporation generally compromises the dynamic tensile strength of the epoxy matrix. Only the moderate 5 wt% loading marginally retains a load-bearing capacity comparable to that of the neat epoxy under low-to-intermediate strain rates. High loadings of 10 wt% and 15 wt% induce marked strength degradation, with the 15 wt% composite suffering the most severe attenuation; however, its dynamic strength rises to 78.49 MPa at 1600 s^−1^, approaching the neat epoxy’s 80.54 MPa, as high-rate loading activates energy dissipation mechanisms such as crazing and shear banding that partially offset the negative effects of agglomeration.

(3) Microscopic fracture analysis reveals a clear evolutionary trend in fracture morphology with increasing nano-Al_2_O_3_ loading. The neat epoxy displays a classic brittle fracture feature characterized by flat cleavage facets and straight fracture steps. With higher filler contents, the fracture surface becomes increasingly rough, with densely distributed tear ridges, microvoids, and craze propagation traces. Crack paths are significantly tortuous due to crack pinning and deflection by well-bonded nano-Al_2_O_3_ particles, and localized matrix plastic tearing is observed around embedded particles. The hybrid fracture configuration with attenuated brittle characteristics and enhanced toughness confirms the effectiveness of nano-Al_2_O_3_ in improving the dynamic fracture resistance of the epoxy matrix when properly dispersed.

Overall, this work quantitatively defines the optimal nano-Al_2_O_3_ loading window for balancing quasi-static and dynamic performance and clarifies the coupled effects of particle agglomeration, interfacial interaction, and strain rate on the deformation and failure behavior of nano-Al_2_O_3_/epoxy composites. The findings provide actionable guidance for the design of nano-reinforced epoxy composites for impact-protective applications, though future work is needed to further optimize dispersion protocols for high-loading systems and quantify the contribution of individual toughening mechanisms.

In comparison with other widely studied epoxy reinforcements, the nano-Al_2_O_3_/epoxy system developed here occupies a distinct niche defined by its specific trade-offs. The principal advantages stem from its balanced functionality: unlike carbon nanotube- and graphene-reinforced epoxies, which typically deliver larger low-loading quasi-static tensile gains but suffer from inherent electrical conductivity and severe high-loading dispersion challenges, the nano-Al_2_O_3_ composites retain ultrahigh volume resistivity (~10^14^ Ω·cm) and relatively high thermal conductivity (~30 W/(m·K)). This combination satisfies the simultaneous demands for electrical insulation and thermal management in protective engineering, outperforming silica nanoparticle systems which are cost-effective but suffer from poor thermal conductivity. Furthermore, the nano-Al_2_O_3_ system exhibits notable high-rate resilience; under dynamic tension at 1600 s^−1^, the 15 wt% composite recovers to 78.49 MPa, approaching the neat epoxy’s 80.54 MPa, demonstrating effective energy dissipation via crazing and shear banding. Conversely, the principal disadvantages lie in its limited quasi-static enhancement and dynamic strength trade-offs. The quasi-static tensile strength gain is modest, with only a 4.0% increase at the optimal 1 wt% loading, and loadings above the 3 wt% critical agglomeration threshold degrade mechanical performance, with the 5 wt% and higher groups falling below the neat matrix under quasi-static tension. Under dynamic tension, nano-Al_2_O_3_ generally lowers tensile strength except for the 5 wt% group at intermediate rates, and even the 15 wt% composite fails to exceed the neat epoxy’s baseline strength. These results indicate that nano-Al_2_O_3_ is best positioned as a balanced-functionality filler for insulating, thermally managed impact-protection applications, rather than as a primary strength-enhancing additive.

## Figures and Tables

**Table 1:** Material parameters for dynamic loading.

$E_{B}$ (Pa)	Density of the Bars ρ (kg/m^3^)	$C_{B}$ (m/s)	Diameter of the Bars d (m)
1.9 × 10^11^	8000	≈4874	0.019
$A_{B}$ **(m^2^)**	**Measurement Uncertainty**	**Gauge Factor**	**Strain Conversion Factor**
2.83 × 10^−4^	0.0995	2.14	0.001

**Table 2:** Testing conditions for dynamic tensile experiments.

No.	Nano-Al_2_O_3_ Content	Gas Pressure (MPa)	Strain Rate (s^−1^)	No.	Nano-Al_2_O_3_ Content	Gas Pressure (MPa)	Strain Rate (s^−1^)	No.	Nano-Al_2_O_3_ Content	Gas Pressure (MPa)	Strain Rate (s^−1^)
1	0 wt%	0.095	600	2	0 wt%	0.12	1100	3	0 wt%	0.15	1600
4	5 wt%	0.095	600	5	5 wt%	0.12	1100	6	5 wt%	0.15	1600
7	10 wt%	0.095	600	8	10 wt%	0.12	1100	9	10 wt%	0.15	1600
10	15 wt%	0.095	600	11	15 wt%	0.12	1100	12	15 wt%	0.15	1600

**Table 3:** Tensile strength (MPa) of nano-Al_2_O_3_/epoxy composites with varying nano-Al_2_O_3_ contents at different strain rates.

	Nano-Al_2_O_3_ Content	0 wt%	1 wt%	3 wt%	5 wt%	10 wt%	15 wt%
Strain Rate							
0.001 s^−1^		43.04 ± 0.33	44.76 ± 0.25	43.4 ± 0.32	39.18 ± 0.22	41.62 ± 0.20	40.54 ± 0.24
0.01 s^−1^		46.12 ± 0.39	48.50 ± 0.30	46.4 ± 0.27	45.72 ± 0.29	41.50 ± 0.31	40.42 ± 0.26

Note: Data are presented as mean ± standard deviation (SD), calculated from *n* = 3 independent specimens.

**Table 4:** Tensile strength (MPa) of nano-Al_2_O_3_/epoxy composites with varying nano-Al_2_O_3_ loadings tested under varying drive gas pressures.

	Nano-Al_2_O_3_ Content	0 wt%	5 wt%	10 wt%	15 wt%
Drive Gas Pressure (Strain Rate: s^−1^)					
0.095 MPa (600)		66.52 ± 0.58	59.91 ± 0.45	66.14 ± 0.49	46.77 ± 0.53
0.12 MPa (1100)		70.11 ± 0.60	64.30 ± 0.52	71.92 ± 0.58	50.92 ± 0.52
0.15 MPa (1600)		80.54 ± 0.55	67.02 ± 0.61	75.85 ± 0.59	78.49 ± 0.48

Note: Data are presented as mean ± standard deviation (SD), calculated from *n* = 3 independent specimens.

## Data Availability

The original contributions presented in this study are included in the article. Further inquiries can be directed to the corresponding author.

## References

[1] Zhou K., Wu Y., Yin L., Luo J., Lu K., Yu B., Shi Y., Zhang S., Jia S. (2025). In situ assembly of polyphosphazene on Fe-MMT nanosheets for high-performance flame-retardant epoxy composites. Polym. Degrad. Stab..

[2] Bian A., Zheng P., Yang Z., Li K., Ai H., Zhou Y., Zhang L., Tang Y., Chow W.K., Liu Q. (2026). Investigation of multi-element modified silicon-based flexible long-chain flame retardant: Synergistic enhancement of flame retardancy and impact toughness of epoxy resin. Polym. Degrad. Stab..

[3] Öztürkmen M.B., Demirel M.Ö., Ağaç Ö., Ece R.E., Öz Y. (2023). Tailored multifunctional nanocomposites obtained by integration of carbonaceous fillers in an aerospace grade epoxy resin curing at high temperatures. Diam. Relat. Mater..

[4] Guadagno L., Pantelakis S., Strohmayer A., Raimondo M. (2022). High-performance properties of an aerospace epoxy resin loaded with carbon nanofibers and glycidyl polyhedral oligomeric silsesquioxane. Aerospace.

[5] Altaf M., Ahmed M.N., Iqbal A., Ali Khan A. (2026). Synergistic effect of CuO-Coated CNTs on the flame retardancy of epoxy resin nanocomposites for aerospace applications. J. Polym. Res..

[6] Yang H., Miao Z., Yang Y., Yan W., Ren L., Yang Z., Guo Y., Yang Y., Wei Y., Tu H. (2025). Enhancing impact resistance of CFRP by incorporating dynamic non-covalent bonds into epoxy resin networks. Compos. Part B Eng..

[7] Meyer O.K., Haney R., Bauder T., Gupta K., Stephanie H., Bordeau J., Wood C., Mintz K., Kumar S., Koerner H. (2025). Multichannel hollow carbon fiber reinforcement in an epoxy resin matrix for direct ink writing of high-performance composites. Mater. Des..

[8] Groh F., Kappel E., Hühne C., Brymerski W. (2019). Investigation of fast curing epoxy resins regarding process induced distortions of fibre reinforced composites. Compos. Struct..

[9] Peerzada M., Abbasi S., Lau K.T., Hameed N. (2020). Additive manufacturing of epoxy resins: Materials, methods, and latest trends. Ind. Eng. Chem. Res..

[10] Tarafdar A., Lin W., Naderi A., Wang X., Fu K., Hosein I.D., Wang Y. (2024). UV-induced frontal polymerization for optimized in-situ curing of epoxy resin for excellent tensile and flexural properties. Compos. Commun..

[11] Reis M.Q.D., Banea M.D., da Silva L.F.M., Carbas R.J.C. (2019). Mechanical characterization of a modern epoxy adhesive for automotive industry. J. Braz. Soc. Mech. Sci. Eng..

[12] Zhang C., Liu Z., Zhang T., Wang X., Yin C., Liu X., Chi Q. (2023). High-temperature resistance and excellent electricalinsulation in epoxy resin blends. J. Appl. Polym. Sci..

[13] Li X., Zhou Y., Bao Y., Wei W., Fei X., Li X., Liu X. (2022). Bismaleimide/phenolic/epoxy ternary resin system for molding compounds in high-temperature electronic packaging applications. Ind. Eng. Chem. Res..

[14] Ding C., Matharu A.S. (2014). Recent developments on biobased curing agents: A review of their preparation and use. ACS Sustain. Chem. Eng..

[15] Feng Y., Hu J., Wang F., Huang Q., Peng C., Xu Z. (2018). Synthesizing promising epoxy acrylate prepolymers applied in ultraviolet cured adhesives based on esterification reaction. Mater. Res. Express.

[16] Rmili W., Deffarges M.P., Chalon F., Ma Z., Leroy R. (2014). Dynamic mechanical properties and thermal effect of an epoxy resin composite, encapsulation’s element of a new electronic component. J. Electron. Mater..

[17] Li Z., Xie K., Huang M., Hu W., Xie Q., Zhu F., Liu J., Li X., Wei W. (2025). Simultaneous toughening and strengthening of epoxy-anhydride thermosets using a glycidyl ether of eugenol-grafted polysiloxane without sacrificing thermal performance. React. Funct. Polym..

[18] Shundo A., Yamamoto S., Tanaka K. (2022). Network formation and physical properties of epoxy resins for future practical applications. JACS AU.

[19] Xian G., Niu Y., Qi X., Tian J., Li C., Yue Q., Guo R. (2024). Water absorption and property evolution of epoxy resin under hygrothermal environment. J. Mater. Res. Technol..

[20] Chen X., Lin Z., Ma T., Gu L., Chen Y., Shi S. (2024). Study on hot-mix epoxy resin based on glass transition temperature and its application for steel bridge deck pavement. J. Mater. Civ. Eng..

[21] Javaid A., Jain D., Kwatra N. (2024). Pre-treatment and its effect on the thermal conductivity of natural lignocellulosic rice straw stubble waste boards: Analysis vis a vis potential for the civil engineering applications. Constr. Build. Mater..

[22] Wang J., Fan K., Du J., Xu J., Dong X., Li X., Ding Y. (2023). Effect of organosilicon modified epoxy resin on slurry viscosity and mechanical properties of polyurethane grouting materials. Constr. Build. Mater..

[23] Zhou Y., Zhang J., Zhang R., Liu E., Wang L., Zhang G. (2023). Strength recovery effect of bisphenol a epoxy resin E44 on rock masses with various crack widths. Adv. Civ. Eng..

[24] Zhang P., Zhang X., Dai X., Wei S. (2026). Individual and synergistic effects of hybrid PVA–steel fiber on mechanical properties of nano-SiO_2_ modified epoxy resin. Gels.

[25] Wijesekara D.A., Sargent P., Ennis C.J., Hughes D. (2021). Prospects of using chars derived from mixed post waste plastic pyrolysis in civil engineering applications. J. Clean. Prod..

[26] Obayashi K., Kojio K. (2025). Adhesive properties of low-cross-linking density cured epoxy resin. Polym. J..

[27] Faggio N., Marotta A., Ambrogi V., Cerruti P., Gentile G. (2023). Fully bio-based furan/maleic anhydride epoxy resin with enhanced adhesive properties. J. Mater. Sci..

[28] Liu C., He Y., Sun M., Zhang X., Zhang B., Bai X. (2023). Influence of epoxy resin species on the curing behavior and adhesive properties of cyanate Ester/Poly(aryl ether nitrile) blends. Polymer.

[29] Han H.-S., Ju K.-S., Pak H.-T., Kyun U., Ri Y.-I. (2024). Improvement of adhesive properties of modified epoxy–novolac resin by acrylonitrile–butadiene rubber grafted poly(chromium methacrylate). RSC Adv..

[30] Yu Y., He K., Hang Z., Liu W., Zhao W. (2024). Effects of adhesive layer thickness on the fracture properties of a concrete-epoxy resin interface. Theor. Appl. Fract. Mech..

[31] Gao Y., Chen K., Zhou B., Wu C., Chen Z., Cen H. (2026). Improvement of epoxy coating chemical stability via fluorinated phenyl isocyanate modification: Substituent-dependent enhancement in anti-corrosion and anti-aging properties. Prog. Org. Coat..

[32] Zhou Q., Lin J., Li Q. (2025). Study of high-strength, low-shrinkage dental resin composites with bifunctional polysilsesquioxane. Dent. Mater..

[33] Li X.-J., Liu C., Zhang C.-Y., Shao Z.-B., Zhao B. (2025). Self-assembly of triazolyl-based cyclomatrix polyphosphazene and melamine cyanurate for flame-retardant, smoke-suppressing, and mechanically robust epoxy resin. Polym. Degrad. Stab..

[34] Jahandideh S., Shirazi M.J.S., Tavakoli M., Mousavi S.M., Hashemi S.A. (2020). High mechanical and thermal performance of Sasobit-modified epoxy resin, prepared via vacuum shock technique. Polym. Test..

[35] Shahabaz S.M., Shetty N., Sharma S., Shetty S.D., Naik N. (2022). Effect of alumina and silicon carbide nanoparticle-infused polymer matrix on mechanical properties of unidirectional carbon fiber-reinforced polymer. J. Compos. Sci..

[36] Kumar K.D., Shantharaja M. (2022). Experimental investigation of Mode-I fracture behavior of nano alumina filler reinforced hybrid composites. Mater. Today Proc..

[37] Kuo M.C., Tsai C.M., Huang J.C., Chen M. (2005). PEEK composites reinforced by nano-sized SiO_2_ and Al_2_O_3_ particulates. Mater. Chem. Phys..

[38] Bian W., Yao T., Chen M., Zhang C., Shao T., Yang Y. (2018). The synergistic effects of the micro-BN and nano-Al2O3 in micro-nano composites on enhancing the thermal conductivity for insulating epoxy resin. Compos. Sci. Technol..

[39] Lin Z., Sun Z., Fu W., Lin Y.-C., Moon K.-S., Wong C.P. (2025). Thermally conductive and electrically insulative alumina/epoxy composites for advanced electronic packaging applications: A comprehensive review of filler morphologies and surface modifications. Mater. Today.

[40] Lim S.H., Zeng K.Y., He C.B. (2010). Morphology, tensile and fracture characteristics of epoxy-alumina nanocomposites. Mater. Sci. Eng. A.

[41] Rajsekhar V., Gattu M. (2023). Size-effect testing: Nano-alumina enhances fracture toughness of epoxy resins. Theor. Appl. Fract. Mech..

[42] Yu J., Huo R., Wu C., Wu X., Wang G., Jiang P. (2012). Influence of interface structure on dielectric properties of epoxy/alumina nanocomposites. Macromol. Res..

[43] Anssari-Benam A. (2024). Hyperinelasticity: An energy-based constitutive modelling approach to isothermal large inelastic deformation of polymers. Part I. J. Mech. Phys. Solids.

[44] Khan A.S., Lopez-Pamies O. (2002). Time and temperature dependent response and relaxation of a soft polymer. Int. J. Plast..

[45] Park H., Cho M. (2020). A multiscale framework for the elasto-plastic constitutive equations of crosslinked epoxy polymers considering the effects of temperature, strain rate, hydrostatic pressure, and crosslinking density. J. Mech. Phys. Solids.

[46] Kumar R., Bhagoria P., Bharadwaj M.R., Tiwari V. (2025). From quasi-static to dynamic: Experimental study of mechanical and fracture behaviour of epoxy resin. Int. J. Impact Eng..

[47] Liu L., Xu K., Xu Y., Zhao Z., Luo G., Chen W. (2022). Experimental study of quasi-static and dynamic tensile behavior of epoxy resin under cyclic hygrothermal aging. Polym. Degrad. Stab..

[48] Cai J., Wei Y., Zhao H., Zhang J., Miao X., Xiao L., Hou L. (2024). Carbon nanotubes grafting aminated epoxy resin with improved elasticity and surface adhesion for enhanced thermal management performance. Colloids Surf. A Physicochem. Eng. Asp..

[49] Santos A.S.D., de Oliveira T.C., Rodrigues K.F., Silva A.A.C., Coppio G.J.L., da Silva Fonseca B.C., Simonetti E.A.N., De Simone Cividanes L. (2021). Amino-functionalized carbon nanotubes for effectively improving the mechanical properties of pre-impregnated epoxy resin/carbon fiber. J. Appl. Polym. Sci..

[50] Yang X., Meng F., Zhang X., Cao B., Fu Y. (2022). Mesoscopic simulation of thermal conductivities of 3D carbon nanotubes, graphene and their epoxy resin based composites. Int. J. Therm. Sci..

[51] Hao Q., Liu S., Wang X., Zhang P., Mao Z., Zhang X. (2024). Progression from graphene and graphene oxide to high-performance epoxy resin-based composite. Polym. Degrad. Stab..

[52] Hussain M.Z., Shah S.Z.H., Megat-Yusoff P.S.M., Choudhry R.S., Ahmad F., Hussnain S.M. (2025). Toughening Epoxy resin system using nano-structured block copolymer and graphene nanoplatelets to mitigate matrix microcracks in epoxy nanocomposites: A DoE based framework. Mater. Today Commun..

[53] Amirova L.M., Khannanov A., Dimiev A.M., Amirov R.R. (2025). The rheology of graphene oxide dispersions in highly viscous epoxy resin: The anomalies in properties as advantages for developing film binders. Liquids.

[54] Ren M., Wanga L., Luoa L., Li T. (2023). Mechanical properties and liquid oxygen compatibility of nano-silica and grapheneoxide modified phosphorus-containing epoxy resin. Mol. Simul..

[55] Jiang Z., Lu P., Zhang R., Bi J., Wang Y., Hu X., Wu J., Wang Z., Li W. (2025). Synthesis and investigation of anti-wear and anti-friction properties in epoxy resin matrix composites filled with nano-silica and basalt flakes. J. Mater. Eng. Perform..

[56] Meng X., Guo W., Sun Y., Zhang C. (2026). Preparation and performance study of nano-silica sol-modified epoxy resin grouting material. Mater. Lett..

[57] Ye Y., Chen H., Wu J., Ye L. (2007). High impact strength epoxy nanocomposites with natural nanotubes. Polymer.

[58] Xu S., Song X., Cai Y. (2016). Mechanical properties and morphologies of carboxyl-terminated butadiene acrylonitrile liquid rubber/epoxy blends compatibilized by pre-crosslinking. Materials.

[59] Chen Y., Li Z., Teng C., Li F., Han Y. (2016). Dielectric properties of polyether sulfone/bismaleimide resin composite based on nanolumina modified by super-critical ethanol. J. Electron. Mater..

[60] Liu M., Zheng K., Liang R., Jiang J., Yang F. (2025). Hybrid alumina-graphene oxide reinforced phenolic friction material. Mater. Today Commun..

[61] Gan L., Liu Y., Yimin Z., Wu J., Lv J., Liu Z. (2025). Fabrication and performance enhancement of wood liquefaction-based carbon fibers modified with alumina nanoparticles. Polymers.

[62] Guessasma S., Belhabib S., Nouri H. (2019). Printability and Tensile Performance of 3D Printed Polyethylene Terephthalate Glycol Using Fused Deposition Modelling. Polymers.

[63] Kolsky H. (1949). An Investigation of the Mechanical Properties of Materials at very High Rates of Loading. Proc. Phys. Soc. Sect. B.

[64] Zhang J.-H., Shang B. (2016). Numerical Study of the Data Processing Methods in SHPB Experiments. Chin. J. High Press. Phys..

[65] Song L., Zhong D. (2023). Stress Wave Separation Based on Standard Hopkinson Pressure Bar Set-up and Unlimited Duration of Experiment Data Processing. Explos. Shock Waves.

[66] Zhang T., Liang J., Wang B., Sun M. (2025). Enhanced mechanical properties of epoxy composites reinforced with silane-modified Al_2_O_3_ nanoparticles: An experimental study. J. Compos. Sci..

[67] Duan Z., He H., Liang W., Wang Z., He L., Zhang X. (2018). Tensile, Quasistatic and Dynamic Fracture Properties of Nano-Al_2_O_3_-Modified Epoxy Resin. Materials.

[68] Zhang H., Zhang H., Tang L., Liu G., Zhang D., Zhou L., Zhang Z. (2010). The Effects of Alumina Nanofillers on Mechanical Properties of High-Performance Epoxy Resin. J. Nanosci. Nanotechnol..

[69] Yazman Ş., Samancı A. (2019). A Comparative Study on the Effect of CNT or Alumina Nanoparticles on the Tensile Properties of Epoxy Nanocomposites. Arab. J. Sci. Eng..

